# Multimodal dynamic response of the *Buchnera aphidicola* pLeu plasmid to variations in leucine demand of its host, the pea aphid *Acyrthosiphon pisum*

**DOI:** 10.1111/j.1365-2958.2011.07760.x

**Published:** 2011-07-28

**Authors:** José Viñuelas, Gérard Febvay, Gabrielle Duport, Stefano Colella, Jean-Michel Fayard, Hubert Charles, Yvan Rahbé, Federica Calevro

**Affiliations:** UMR203 BF2I, Biologie Fonctionnelle Insectes et Interactions, INSA-Lyon, INRA, Université de LyonBât. Louis Pasteur, 20 av. Albert Einstein, F-69621 Villeurbanne, France

## Abstract

Aphids, important agricultural pests, can grow and reproduce thanks to their intimate symbiosis with the γ-proteobacterium *Buchnera aphidicola* that furnishes them with essential amino acids lacking in their phloem sap diet. To study how *B. aphidicola*, with its reduced genome containing very few transcriptional regulators, responds to variations in the metabolic requirements of its host, we concentrated on the leucine metabolic pathway. We show that leucine is a limiting factor for aphid growth and it displays a stimulatory feeding effect. Our metabolic analyses demonstrate that symbiotic aphids are able to respond to leucine starvation or excess by modulating the neosynthesis of this amino acid. At a molecular level, this response involves an early important transcriptional regulation (after 12 h of treatment) followed by a moderate change in the pLeu plasmid copy number. Both responses are no longer apparent after 7 days of treatment. These experimental data are discussed in the light of a re-annotation of the pLeu plasmid regulatory elements. Taken together, our data show that the response of *B. aphidicola* to the leucine demand of its host is multimodal and dynamically regulated, providing new insights concerning the genetic regulation capabilities of this bacterium in relation to its symbiotic functions.

## Introduction

Aphids are one of the most important pests of agricultural plants. An interesting physiological feature of this insect group is its dependence on symbiotic microorganisms of the *Buchnera* genus for growth and reproduction. This symbiosis, established some 80–150 million years ago (Moran *et al*., 1993; Von Dohlen and Moran, 2000), has given rise to a close relationship between the two members of the association. *Buchnera aphidicola* furnishes its host with essential amino acids that the insect cannot find in sufficient amounts in its exclusive diet of phloem sap, and the aphid supplies the amino acids and core metabolites that the symbiont cannot synthesize anymore due to the loss of its central metabolic pathways (Brinza *et al*., 2009; Wilson *et al*., 2010).

The composition of phloem sap is not constant and can vary widely in response to environmental cues. It has been shown to change between day and night in different plant species (Smith and Miburn, 1980; Winter *et al*., 1992; Gholami *et al*., 2004) and, in a more subtle way, also during the diurnal cycle (Sharkey and Pate, 1976) or according to the host plant developmental stages (Pate *et al*., 1998). Recently, analysis on wheat sieve tubes has clearly shown variations in the concentration of several amino acids, in particular that of leucine, during the diurnal cycle (Gattolin *et al*., 2008). These observations give rise to the question of the ability of the aphid/*Buchnera* symbiotic system to modulate its amino acid biosynthesis in response to such changes in phloem sap amino acid concentrations.

Although several biochemical studies, performed on artificial diets at known concentrations of amino acids, have shown the capability of the system, and particularly that of *B. aphidicola*, to respond to changing environmental conditions (Douglas, 1988; Douglas and Prosser, 1992; Febvay *et al*., 1995; 1999; Liadouze *et al*., 1995; Sasaki and Ishikawa, 1995), the regulation properties of the highly reduced genome of this bacterium are still unknown. From 2000 to 2006, four genomes of *B. aphidicola,* isolated from different aphid species, were sequenced (Shigenobu *et al*., 2000; Tamas *et al*., 2002; van Ham *et al*., 2003; Pérez-Brocal *et al*., 2006). In 2009, seven additional genomes from the pea aphid species were published (Moran *et al*., 2009). All the *Buchnera* genomes share similar properties with other endosymbiont genomes: (i) a lack of recombination, (ii) an increase in the nucleotide substitution rate, (iii) a bias towards AT base pairs sequence content, (iv) a loss of standard codon usage bias, (v) a fixation of deleterious mutations by random genetic drift (Wernegreen, 2002) and, above all, (vi) an extremely reduced genome size, ranging from 0.42 to 0.65 Mb. Despite this shrinkage, the *B. aphidicola* genome has conserved most of the genes involved in the biosynthesis of essential amino acids, while losing almost all the genes regulating their expression (Shigenobu *et al*., 2000; Moran *et al*., 2005; Moran and Degnan, 2006). This loss of transcriptional regulators, coupled with an alteration in the usual transcriptional regulatory mechanisms present in relative free-living bacteria (i.e. the loss of leader peptide coding regions and, thus, of attenuation mechanisms, or the evolution of DNA regulatory sequences imposed by the high AT bias of the *B. aphidicola* genomes), strengthens the hypothesis that the capability of this bacterium to regulate its gene expression, and so respond to its aphid host needs, is degenerating. Regarding, in particular, amino acid production, it has been proposed that novel regulatory mechanisms may have been selected in the context of symbiosis-imposed genome reduction in *B. aphidicola* (Moran *et al*., 2003), but no additional specific data have been produced to support this hypothesis. The conservation of a basal transcriptional organization (conservation of a correlation between gene expression profiles and genome organization and of the organization of genes in transcription units) has been observed in this bacterium (Viñuelas *et al*., 2007; Brinza *et al*., 2010). Nevertheless, microarray analyses of the response of *B. aphidicola* to amino acid variations in the aphid diet have shown the absence of a clear correlation between the symbiont transcriptional response and the scaling of essential amino acid biosynthesis to the aphid demand (Moran *et al*., 2003; 2005; Charles *et al*., 2006; Reymond *et al*., 2006).

Despite their important size reduction, a functional property of the *B. aphidicola* genomes is the existence of one or two plasmids (pTrp and pLeu) carrying genes specific for tryptophan and leucine biosynthesis, probably translocated from the bacterial chromosome during the adaptation to intracellular life (Latorre *et al*., 2005; Gil *et al*., 2006). One unique feature of the pLeu plasmid is the presence of all the genes encoding the enzymes specific to the leucine biosynthesis pathway present in the *B. aphidicola* genome, whereas only two genes coding for the two subunits of an enzyme in the tryptophan biosynthesis pathway are located on the pTrp plasmid. Contrasting with the genomic stasis of the *B. aphidicola* chromosome for the last 50 million years (Tamas *et al*., 2002), the comparison of these two plasmids of symbionts from different aphid species suggests the existence of gene plasticity including, for example, rearrangements and chromosomal back-transfers (Gil *et al*., 2006). It is noteworthy that such small plasmids containing nutrition-related genes (i.e. encoding anabolic enzymes, and apparently devoid of active recombination properties) are unique in the bacterial world. The selective pressure responsible for their persistence seems, therefore, to originate strictly from their trophic role in symbiosis.

To date, the maps of the pLeu plasmids have been described in four aphid subfamilies: the Aphidinae, the Pterocommatinae, the Thelaxinae and the Lachninae (Bracho *et al*., 1994; van Ham *et al*., 1997; Baumann, 2005; Latorre *et al*., 2005), with a size ranging from 6.5 to 8.5 kb. The minimal gene set of the pLeu plasmid is composed of the *leuABCD* genes together with one or two genes encoding an incFII-type replicase (*repA*). In the Aphidinae, Pterocommatinae and Thelaxinae subfamilies, the *B. aphidicola* pLeu plasmid also carries either an *ibp* (encoding a heat-shock protein) or, more commonly, a *yqhA* coding sequence (encoding a putative membrane protein). The chromosomal location of the leucine cluster, only found in the Pemphiginae and the Chaitophorinae subfamilies, seems not to be of ancestral origin and a plausible explanation for this location is based on back-transfers to the chromosome by recombination scenarios (Sabater-Muñoz *et al*., 2004). In addition to the plasticity of the pLeu map between the different aphid subfamilies, some studies have shown a variability of the pLeu copy number between different aphid species. For example, keeping in mind that *B. aphidicola* is a bacterium harbouring a highly polyploid genome, the symbionts from *Diuraphis noxia* and *Uroleucon ambrosiae* contain, respectively, 0.9 and 1.6 copies of pLeu copy number per chromosome unit (Thao *et al*., 1998; Plague *et al*., 2003), whereas 23.5 times more pLeu plasmid than chromosome copy number were found in *Schizaphis graminum* (Thao *et al*., 1998). It has been suggested that this variability among aphid species could be related to the metabolic requirements of the aphid when adapting to different host plants (Thao *et al*., 1998).

The existence of the pLeu plasmid, and its conservation during the course of evolution and across different aphid-host species, suggests that leucine may be an amino acid of particular importance in aphid symbiosis, as compared with the other amino acids whose biosynthetic genes are located on the chromosome. The latter hypothesis was only partially confirmed in the *Aphis fabae* symbiosis, where it was found that leucine is the amino acid for which the contribution of *Buchnera*-derived amino acids to net protein growth of the symbiotic aphids is the most important (Douglas *et al*., 2001). Further, studies addressing the question of the specific physiological importance of leucine for aphids are lacking. The location of the leucine biosynthesis genes on a plasmid has led to hypothesize the evolution of novel mechanisms to control the regulation of these genes in response to the aphid metabolic demand (Moran *et al*., 2003). Nevertheless, to our knowledge, only Moran and colleagues (2005) have analysed the global transcriptional response (including pLeu genes) to a moderate leucine excess in the *S. graminum* symbiosis, but no study has involved a functional analysis of the complete genetic response of the *B. aphidicola* pLeu plasmid to specific leucine starvation or excess.

In this work, we have combined aphid physiology analyses with metabolic and molecular approaches in order to elucidate: (i) the importance of leucine for the pea aphid *Acyrthosiphon pisum* and (ii) the capability of the pLeu plasmid to be regulated by the leucine demand of the aphid host. First, an analysis of the aphid biological response to variations in leucine dietary concentrations allowed us to establish that leucine is a limiting factor for aphid growth and that it has a stimulatory feeding effect on the pea aphid, conversely to the other branched-chain amino acids valine and isoleucine. We then quantified the *B. aphidicola* leucine biosynthesis from its prominent natural precursor, sucrose, using ^14^C tracing, thereby demonstrating that the symbiont is able to supply and modulate its leucine production according to aphid demand. The molecular analyses presented here clearly show that, despite its highly reduced genome, *B. aphidicola* is able to perceive a leucine stress imposed on its host from the first 12 h of treatment, and this generates a multimodal dynamic response. A short-term response (12–48 h after the beginning of the treatment) involves a strong regulation of the mRNA levels of all the pLeu located genes. A medium-term (48–72 h) response involves a moderate modification of the pLeu copy number. All these results are analysed in the light of a renewed annotation of potential regulatory elements located in the pLeu plasmid and of the experimental confirmation of the *leuABCD* operon in *B. aphidicola*.

## Results

### Aphid growth performance and behaviour

**Fig. 1:**
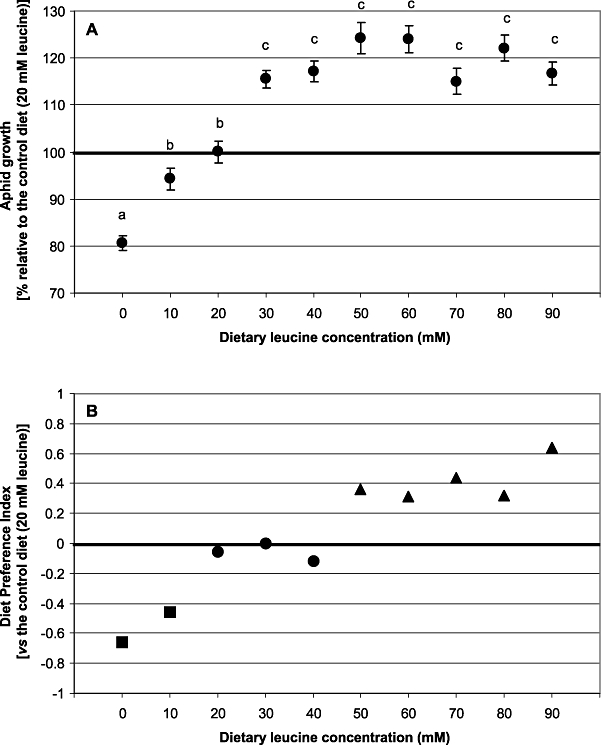
Effect of dietary leucine concentration on pea aphid (*A. pisum*) performance and behaviour. A. Aphid growth for different leucine concentrations in the diet. The results (means ± SE, *n* = 54) are expressed as a percentage of the aphid growth on the 20 mM leucine control diet and compared using a Tukey–Kramer HSD test. Conditions with different letters show statistically different means at 95% confidence level. B. The behavioural response of *A. pisum* to variations in dietary leucine concentration has been evaluated in choice tests, comparing the different tested concentrations with the control diet. Diet Preference Index (DPIx) quantifies the choice of test diet over the control (1 is complete preference of the tested diet over the control, −1 is the reverse). Statistical significance is given following a Wilcoxon non-parametric signed rank test testing DPIx = 0 at 95% confidence level: • no significant difference between the observed DPIx and 0; 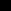 DPIx lower than 0; ▴ DPIx higher than 0. Similar assays with valine and isoleucine showed no significant choice for these amino acids.

The effect of leucine dietary concentrations on aphid growth performance is shown in Fig. 1A. The aphid weight for the different leucine concentrations is reported as the percentage of the weight obtained for aphids reared on the control diet (20 mM leucine). A significant effect of leucine concentration on pea aphid growth was observed (anova*F*-test, *P* < 10^−4^). More precisely, a reduction of 20% in the aphid weight was found when insects were reared on a leucine-depleted diet and, conversely, a 20% increase in the aphid body weight was observed for diets with a leucine concentration higher than 20 mM.

Binary choice tests were subsequently used to analyse the pea aphid feeding behaviour when facing dietary variations in leucine levels (Fig. 1B). A significant positive correlation between the insect diet preference and leucine concentration in the nutritional medium was found (anova*F*-test, *P* < 10^−4^). Compared with the 20 mM leucine reference diet, we found that artificial diets with a leucine concentration lower than 20 mM and higher than 40 mM were significantly less or more readily selected by the insects respectively (Wilcoxon non-parametric signed rank test, *P* < 0.05). Importantly, this stimulatory effect of leucine on the aphid's choice of diet was only seen for leucine and not for the other two branched-chain essential amino acids, valine and isoleucine (data not shown).

### Amino acid neosyntheses in symbiotic pea aphid

**Fig. 2:**
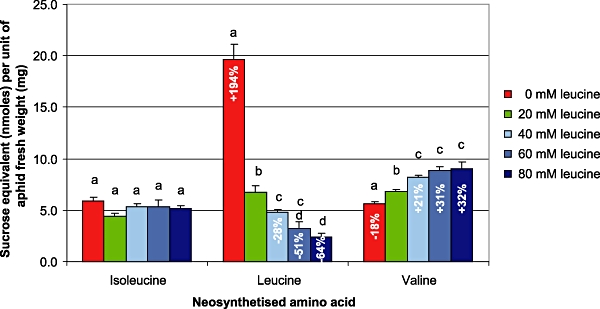
Neosynthesis of branched-chain amino acids from sucrose in symbiotic pea aphid reared on diets with different leucine concentrations. Values (means ± SE, *n* = 5) are expressed in sucrose equivalent (nmol) per unit of aphid fresh weight (mg). Means comparisons were performed with a Student–Newman–Keuls test. For each amino acid, conditions with different letters show statistically different means at 95% confidence level. For each significant response, the percentage of variation relative to the control diet is indicated.

To analyse the metabolic response of *B. aphidicola* to variations in the leucine demand of its aphid host, we quantified the amino acid biosyntheses in symbiotic aphids reared during the entire span of their larval development (7 days) on artificial diets containing different leucine concentrations (0–80 mM). These biosyntheses – from an external carbon source (sucrose) and quantified using ^14^C labelling – are carried out by the symbiotic bacterium for essential amino acids and by the insect host for the non-essential ones (Shigenobu *et al*., 2000; Wilson *et al*., 2010; and Fig. S1). The neosyntheses of all amino acids, expressed in sucrose equivalent (nmol mg^−1^ of fresh aphid mass), are reported in Table S1. In Fig. 2, we focus on the symbiont-driven biosynthesis of the three branched-chain essential amino acids. The symbiotic aphid was able to respond in a significant way to variations in dietary leucine by changing the amount of neosynthesized leucine (anova*F*-test, *P* < 10^−3^). Leucine neosynthesis was significantly enhanced (+194%, if compared with the control diet) when this amino acid was absent from the aphid diet, increasing from 6.7 to 19.7 nmol sucrose equivalent mg^−1^ of aphid fresh weight (Student–Newman–Keuls test with significant *post hoc* groups, *P* < 0.05). Since the six carbons of leucine (synthesized directly from pyruvate, the end product of glycolysis) come from glucose (Macdonald *et al*., 2011), the 19.7 nmol of sucrose equivalent mg^−1^ of aphid fresh weight produced by the symbiotic aphid, following leucine depletion, correspond to 39.4 nmol of neosynthesized leucine per mg of aphid fresh weight. In contrast, leucine biosynthesis significantly decreased, by an average of 48%, for diets with a leucine concentration higher than 20 mM. Leucine dietary supply had an opposite effect on valine biosynthesis (anova*F*-test, *P* < 10^−3^): the valine neosynthesis decreased following leucine nutritional depletion and increased in conditions of leucine excess (Student–Newman–Keuls test, *P* < 0.05). Finally, no significant effect of dietary leucine concentration was observed on isoleucine neosynthesis by *B. aphidicola* (anova*F*-test, *P* = 0.14). Leucine dietary supply had no effect on almost all the other individual amino acid neosyntheses (Table S1); only threonine, lysine (both essential amino acids) and tyrosine (an important aromatic metabolite) were significantly increased with the augmented supply of dietary leucine, this being most probably due to an increased growth-related effect.

### Regulation of the *B. aphidicola* pLeu plasmid after 7 days of treatment

To understand the molecular basis of the adaptive response of the pea aphid/*Buchnera* coupled metabolism to the strength of the nutritional demand, we analysed the molecular response of the pLeu plasmid in conditions of leucine starvation (0 mM leucine) or excess (60 mM leucine), relative to the control diet (20 mM leucine), by quantifying: (i) the transcript levels of all the genes located on pLeu and involved in leucine biosynthesis and (ii) the pLeu plasmid copy number. As for our previous metabolic analyses, we performed the tests after 7 days of treatment, which corresponds to the entire aphid larval life cycle. Despite the highly significant response observed for leucine neosynthesis (Fig. 2), this long-term analysis did not show any significant genetic regulation of the pLeu plasmid replication or transcription at the end of the treatment period (data not shown).

### Kinetic analysis of the transcription of *B. aphidicola* pLeu genes

**Fig. 3:**
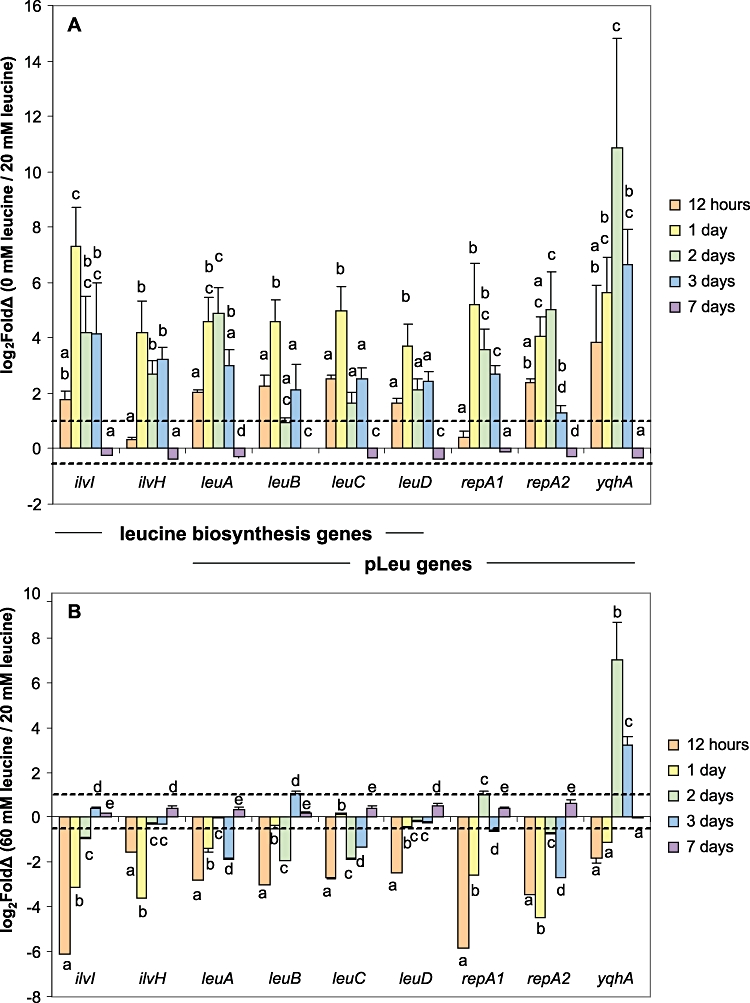
Transcriptional response of *B. aphidicola* to variations of leucine concentration in the aphid diet. A. Differential gene expression variation (log_2_FoldΔ, means ± SE, *n* = 3) of genes involved in leucine biosynthesis and/or located on the pLeu plasmid for aphids reared from 12 h to 7 days on a medium completely deprived of leucine (0 mM leucine), relative to the control diet (20 mM leucine). The thresholds of significant biological differentially expressed genes are fixed, respectively, at 1 and −0.5 for over- and under-expression. Means comparisons were performed with a Tukey–Kramer HSD test. For each gene, time point responses with different letters show statistically different means at 95% confidence level. B. The same as in (A), but for aphids reared from 12 h to 7 days on a medium containing excess leucine (60 mM leucine), relative to the control diet (20 mM leucine).

Following these preliminary results at 7 days, a time-course experimental plan was performed to analyse the genetic response of *B. aphidicola* to aphid dietary leucine starvation or excess, with aphids reared for 12 h, 1, 2, 3 or 7 days on artificial diets containing 0, 20 or 60 mM leucine. We quantified the mRNA levels of the seven pLeu genes and we also included two supplementary *B. aphidicola* chromosomal genes involved in the leucine biosynthetic pathway, the *ilvI* and *ilvH* genes (see the pathway in Fig. S1). The differential gene expression variations for each gene after normalization are shown in Fig. 3. Under the leucine starvation condition (Fig. 3A), a global and significant mRNA overexpression was observed starting 12 h after the beginning of the treatment. For the leucine biosynthetic genes (chromosomal *ilvIH* and plasmidic *leuABCD*), the overexpression was maximal at day 1 and then started to decrease from day 2, except for *leuA*, for which the overexpression persisted for one more day. The response of all these genes to leucine starvation was no longer significant after 7 days. A similar profile was observed for the three other pLeu genes not involved in the leucine biosynthetic pathway (*repA1*, *repA2*, *yqhA*): they were upregulated between 12 h and 2 days of treatment and their response started to decrease from day 3.

A specific transcriptional response for the biosynthetic leucine genes *ilvIH* and *leuABCD* was also seen when the aphid artificial diet was enriched in leucine (Fig. 3B). In this case, a fast and significant downregulation of these genes appeared after 12 h of treatment. This downregulation, relative to the response observed at the beginning of the treatment, persisted for some genes for 1 or 2 days and it became non-significant at day 3 for all the genes analysed, with the exception of *leuA* and *leuC*. A fairly similar response was observed for the *repA1* and *repA2* genes. Interestingly, the *yqhA* transcriptional response to leucine excess consisted of a downregulation at 12 h and after 1 day of treatment, and the gene was suddenly overexpressed when the treatment with 60 mM leucine was continued for 2 or 3 days. Finally, as for the leucine starvation condition, and as previously observed in this work for all the analysed genes, no significant variation in gene transcription regulation was observed for the *yqhA* gene after 7 days of treatment.

### Kinetic analysis of pLeu copy number

**Fig. 4:**
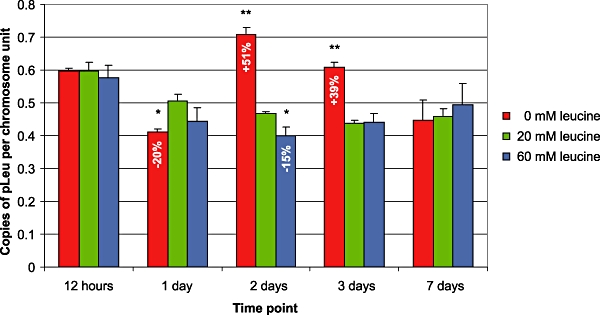
*B. aphidicola* pLeu plasmid copy number calculation in response to variations of leucine concentration in the aphid diet. For each time point (from 12 h to 7 days) the pLeu copy number per chromosome unit (means ± SE, *n* = 9) measured for the depleted (0 mM leucine) and for the excess condition (60 mM leucine) is compared with the values obtained on the reference diet (20 mM leucine) using a Dunett's test. Statistically different values are marked with ‘**’ or ‘*’, indicating a 99% or a 95% confidence level respectively. For each significant response, the percentage of variation relative to the control diet is indicated.

The same time-course experimental plan (12 h to 7 days) was reproduced to analyse the leucine plasmid copy number variations in response to leucine starvation/excess in the aphid host diet. Figure 4 shows the pLeu copy number normalized per chromosome unit for the three selected dietary conditions and for the five time points used throughout this work. The basal pLeu copy number per chromosome unit for the 7-day-old aphids reared on artificial control medium was 0.49 ± 0.03. This agrees with data from Moran and colleagues (Moran *et al*., 2003) who quantified, at 0.6, the *B. aphidicola* pLeu copy number per chromosome unit from another *A. pisum* clone. No significant variation in pLeu copy number was observed for aphids reared for 12 h on diets with no leucine, or with an excess of this amino acid. However, a moderate but significant response of the symbiont was observed, with an increase in the pLeu copy number of, respectively, 51% and 39% when compared with the reference diet, 2 or 3 days after the beginning of the starvation period (Dunett's test, *P* < 10^−2^). In line with this upregulation, a slight decrease of 15% in the pLeu copy number, relative to the control condition, was observed after 2 days on the diet with an excess of leucine (Dunett's test, *P* < 0.05). Paradoxically, we observed a significant reduction in the pLeu copy number after 1 day of leucine starvation (Dunett's test, *P* < 0.05). As for transcriptional regulation, after 7 days on artificial diets containing 0, 20 or 60 mM leucine, no significant variation in plasmid copy number was detected between treatments.

### Reappraisal of the *B. aphidicola* pLeu plasmid annotation and experimental validation of the *leuABCD* operon

**Fig. 5:**
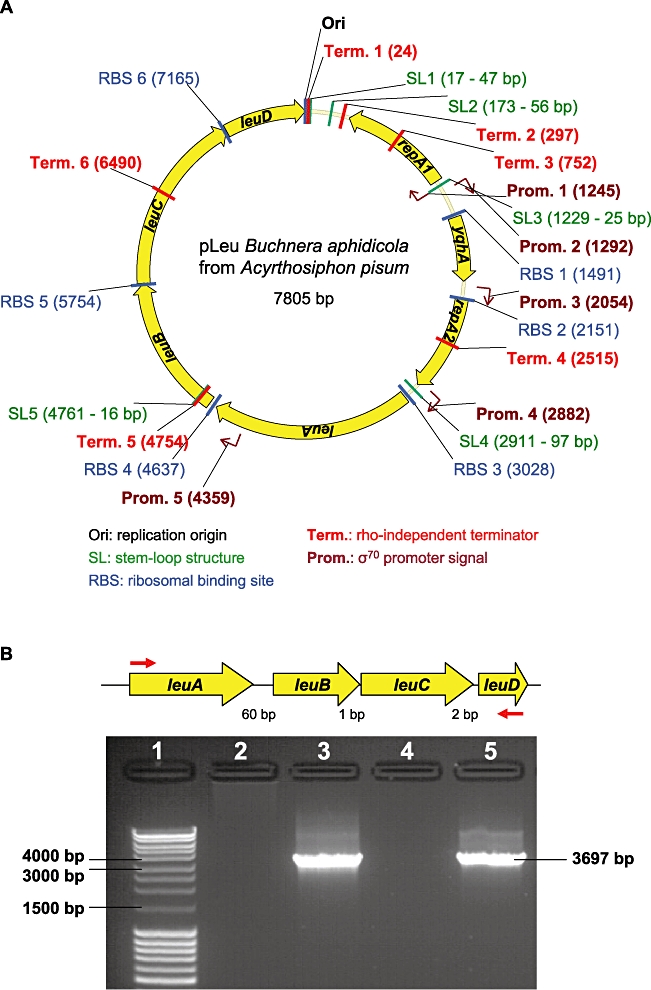
Re-annotation of the pLeu plasmid map and experimental validation of the polycistronic *leuABCD* structure in *B. aphidicola* from the pea aphid. A. A new bioinformatic analysis of the pLeu sequence allowed us to localize the following putative regulatory elements: stem-loop structures are in green (SL), promoter sequences in brown (Prom.), rho-independent terminators in red (Term.), ribosomal binding sites in blue (RBS). The distance of the first nucleotide of these putative elements from the pLeu ori, as well as the length in base pairs for the stem-loop structures, are indicated in brackets. B. The presence of a polycistronic structure for the *leuABCD* genes in the pLeu plasmid was confirmed by RT-PCR. Lane 1, Fermentas 10 kb DNA Ladder mix; lane 2, negative control (water); lane 3, PCR positive control on genomic DNA; lane 4, negative control of RT-PCR (PCR reactions were performed in the absence of Reverse Transcriptase); lane 5, RT-PCR products. Red arrows on the *leuABCD* operon scheme indicate primer positions.

A summary of the re-annotation of the *B. aphidicola* pLeu plasmid is given in Fig. 5A (and detailed in Fig. S2). All the plasmidic genes, with the exception of *repA1*, were found to display a canonical ribosomal binding site (RBS) that, in the context of *B. aphidicola* evolution, probably indicates selection for the conservation of high expression of these genes. Promoter signals (i.e. σ^70^ binding sites, Fig. S2) were found upstream of the *repA1*, *yqhA*, *repA2* and *leuA* sequences, and for a potential *leuB* alternative promoter (within the *leuA* coding sequence). A comparative evolutionary analysis performed on 10 aphid species belonging to two tribes of the Aphididae family, the Aphidini and Macrosiphini tribes, which diverged more than 50 million years ago, allowed us to strengthen our promoter predictions for the pLeu genes (Fig. S3). Indeed, promoters were detected for nine and for 10 out of the 10 species, respectively, for the *repA2* and *leuA* genes. Sequence information on *yqhA*, *repA1* and *leuB* promoters was available only in four aphid species: strong promoter signals were found for the *yqhA* and *repA1* genes (in the four aphid species) whereas conservation was weaker for the *leuB* gene.

The most significant inverted repeat structures were identified as two rho-independent terminators for the *leuD* and *repA1* genes, both situated in the non-coding region around the origin of replication, together with a strikingly well-conserved stem-loop structure of 97 nucleotides located downstream of the *repA2* sequence and upstream of the putative *leuABCD* operon. The bioinformatic analysis also revealed the existence of a stem-loop structure at the beginning of the *leuB* coding sequence.

Finally, due to the important short-distance syntenies between the *B. aphidicola* and *Escherichia coli* genomes, coupled with the conservation of the *leuABCD* gene organization along the pLeu plasmid during aphid evolution, the operonic organization of the *leuABCD* genes has always been supposed, but never before demonstrated, in the pea aphid symbiont. Figure 5B shows the RT-PCR results confirming the existence of a *leuABCD* polycistronic structure in *B. aphidicola* from the pea aphid. The conservation of this operonic structure, despite the important reduction in the *B. aphidicola* genome size and the relocation of the *leuABCD* genes from the chromosome to a plasmid, indicates a key role for these genes in aphid symbiosis.

## Discussion

Leucine is one of the 10 essential amino acids that aphids cannot produce themselves nor find in sufficient quantity in their strict phloem sap diet. In this study we demonstrate the unique role of this amino acid in the pea aphid/*Buchnera* symbiosis. First, leucine is a limiting factor for aphid growth and it is a significant phagostimulant to *A. pisum*. Leucine is the only branched-chain amino acid having this stimulatory effect on the pea aphid, which could be taken as evidence of the existence of selective pressures on this trait. We also observed a specific metabolic tuning of leucine biosynthesis by aphids with their symbiotic bacteria in response to variations of leucine concentration in the aphid diet. As confirmed by the genome sequence analyses of *B. aphidicola* (Shigenobu *et al*., 2000) and of the pea aphid (International Aphid Genomics Consortium, 2010; Wilson *et al*., 2010), the biosynthesis of this essential amino acid is under the control of the bacterium, with the exception of the last step (Fig. S1). To our knowledge, this is the first study showing a significant adjustable and specific metabolic response of *B. aphidicola* to a single amino acid dietary stress imposed on the aphid, thus indicating the crucial role of the *Buchnera*-derived leucine for pea aphid development and growth. Our ^14^C tracing data also demonstrate that leucine concentration in the aphid diet specifically impacts the neosynthesis of valine but does not induce significant variations in isoleucine biosynthesis. In particular, the system reduces valine biosynthesis in the case of leucine depletion and increases it in the case of leucine excess. With regard to the branched-chain amino acid superpathway, these results suggest that the response of *B. aphidicola* to leucine concentration in the aphid diet is specifically targeting the leucine and valine, leaving isoleucine under the upstream (aphid) control of the 2-oxobutanoate pools, and valine under the competing effect of leucine streams for consumption of 2-oxoisovalerate (Fig. S1, and see Umbarger, (1987) for a review on the branched-chain amino acids superpathway in Enterobacteria). Apart from this specific effect on valine neosynthesis, the leucine dietary depletion had no effect on the intensity of all the other amino acid biosyntheses (Table S1). However, excess leucine in the aphid diet also activated the neosyntheses of tyrosine, threonine and lysine. It is noteworthy that threonine, phenylalanine (the unique tyrosine precursor), leucine and lysine have been identified as the most exported amino acids by *B. aphidicola* to the pea aphid: 50% of Thr, 47% of Phe and Leu, and 43% of Lys, synthesized by the symbiotic bacterium, are exported to the host in this symbiosis (Thomas *et al*., 2009). In fact, higher leucine production leads to an increase in aphid protein biosynthesis, which necessitates the availability of all the proteinogenic amino acids, especially those mentioned above, which are derived from the bacterium and whose dietary supply is probably limited in the context of the artificial diet used here for the rearing of aphids.

Our study also provides new insights into the genetic regulation capabilities of *B. aphidicola* in relation to its symbiotic functions. We found that a specific regulation of the pLeu plasmid of this symbiotic bacterium occurs in response to leucine starvation or excess in the aphid diet. This regulation involves an early important transcriptional regulation (starting 12 h after the beginning of the treatment, Fig. 3), followed by a slight modification of the pLeu copy number (Fig. 4). No genetic regulation was observed after 7 days of treatment.

All the genes involved in leucine biosynthesis tested here were significantly overexpressed in the leucine deficit conditions and, conversely, under-regulated in conditions of leucine excess. The consequence of this early genetic regulation is probably a modulation of the activity of the key enzymes involved in leucine biosynthesis, and this results in variations of leucine production by the symbiotic system in response to the diet constraints. If we take into account the results regarding the leucine depletion conditions, we can hypothesize that the important short-term gene expression regulation could lead to an accumulation of biosynthetic enzymes with a consequently enhanced production of leucine precursors and/or leucine. The result of this accumulation is that, after 7 days of treatment (this being the time point at which we were able to obtain metabolic data) the genetic regulation is no longer necessary, even if its effect on leucine neosynthesis is still observable. The cumulative production of leucine in the depleted diet, that we estimated to be 39.4 nmol mg^−1^ of aphid fresh weight, is very close to the total leucine levels in aphids reared on plant or optimal artificial diets at the end of larval development, estimated to be between 40 and 45 nmol mg^−1^ of aphid fresh weight (Febvay *et al*., 1988; 1999;). Therefore, even in the complete absence of leucine in the diet, the cumulative production of this amino acid by the symbiotic bacterium, throughout larval development, should almost entirely satisfy the needs of the host for optimal growth.

Furthermore, in the leucine depletion conditions, the kinetic profiles of all the leucine biosynthetic genes were very similar, with the exception of the *leuA* gene whose overexpression persisted for an extra day, compared with the other leucine biosynthetic genes. The 2-isopropylmalate synthase (EC 2.3.3.13), encoded by *leuA,* operates at the branching-point of the leucine/valine pathways and catalyses the conversion of 2-oxoisovalerate to 2-isopropylmalate (Fig. S1). In the case of leucine privation, an increase in this enzyme activity, resulting from a *leuA* overexpression, could lead to the use of 2-oxoisovalerate for leucine biosynthesis at the expense of valine production. Recent work, performed in *E. coli*, has shown that l-valine production is directly enhanced by increasing the carbon flux towards this amino acid, through the availability of the precursors pyruvate and 2-oxoisovalerate, and this highlights the critical role of the *leuA* gene in the superpathway: by knocking out this gene a significant increase in 2-oxoisovalerate availability and, therefore, in l-valine synthesis was observed (Park *et al*., 2007). Based on our data, it is tempting to suggest that the orientation of 2-oxoisovalerate towards leucine synthesis, by LeuA, could be one of the key steps for leucine production regulation in response to the aphid demand in *B. aphidicola*. Nevertheless, in the context of the pea aphid/*Buchnera* symbiosis, we cannot exclude the possibility that other regulatory mechanisms (enzymatic activity or flux control by the host) could add their effect to the LeuA action in order to control the balance in the leucine/valine biosynthesis, as suggested by the fact that the valine neosynthesis reduction we reported in this work was not quantitatively correlated to the leucine production increase following leucine depletion.

Apart from the leucine biosynthetic genes, we also observed an important overexpression of the *yqhA* gene in response both to leucine starvation and excess, although the kinetics of the induction for the two conditions appeared to be different. The YqhA protein in *B. aphidicola* is a homologue of the corresponding protein of *E. coli*, the proteins from the two organisms showing 60% of identity and 88% of similarity in their amino acid sequences respectively (data not shown). In *E. coli*, YqhA has been annotated as a putative membrane protein and a global topology analysis experimentally assigned this protein to the inner membrane proteome (Daley *et al*., 2005). The drastic transcriptional upregulation of the *yqhA* gene in response to a leucine stress, and its high conservation in the leucine plasmids of the Aphididae family (94–96% of amino acid sequence identity in this aphid group, data not shown), strongly supports the hypothesis that this protein could play a key role in the response of aphid/*Buchnera* symbiosis to a leucine nutritional stress.

Gene expression studies have shown that the transcriptional response of free-living bacteria to a nutritional stress is dynamic, taking place within a few minutes or hours after the beginning of the treatment, and it can be clearly characterized using time-course analyses, the duration of which differs according to the effect (phenotype) being analysed and the bacterial physiology (for examples see Khodursky *et al*., 2000; Chang *et al*. 2002; Durfee *et al*. 2008). The choice of appropriate treatment times and experimental points in a time-course analysis of gene expression response in non-cultivable symbiotic bacteria is difficult to establish because the symbionts cannot be treated outside of their host and, hence, their environment is highly dependent on the host homeostasis and physiology. Indeed, prior to this work, no information was available on the delay that exists between the beginning of treatment on the insect and the stress perception by the symbiont in the pea aphid/*Buchnera* symbiosis. The majority of studies, analysing the transcriptional response of *B. aphidicola* to a nutritional stress imposed on the aphid host, focused on long-term treatments (i.e. 7 days). In agreement with the results presented here, these previous studies have reported the absence of a specific transcriptional response of the *B. aphidicola* genes (including pLeu genes) involved in the corresponding pathways after 7 days of essential amino acid depletion (Charles *et al*., 2006; Reymond *et al*., 2006). The first study that takes into account the short-term response of this symbiotic bacterium to nutritional stresses was published by Moran and colleagues (Moran *et al*., 2005). They analysed the global transcriptomic response of *B. aphidicola* from 5-day-old aphids (*S. graminum* and *A. pisum* species) either left for a further 24 h without any amino acid supply (global amino acid starvation) or placed on barley leaves inserted in solutions containing an individual amino acid excess. The authors reported the presence of a slight transcriptional regulation in response to the imposed stress. In particular, contrary to the results obtained in our study of the pea aphid symbiont where the peak of the transcriptional regulation of leucine biosynthetic genes was observed after 12–24 h of treatment, Moran and colleagues reported the absence of a specific response of the symbiont of *S. graminum* to a leucine excess (42 mM), using comparable time points. These differences in the results might be due to the age of aphids used in the two studies: a leucine stress can be much more effective on neonate aphids (and their symbionts), such as the ones used in our study, than on the almost adult aphids used by Moran and colleagues. Furthermore, we cannot exclude the possibility that these differences could also be dependent on the type of stress we imposed on the aphids. The concentration of 60 mM, used for a leucine excess in our transcriptional study, would be expected to have a bigger impact on the symbiotic system than the 42 mM concentration used by the Moran group. This interpretation is indirectly supported by the considerable difference we observed between the 40 mM and 60 mM Leu concentrations, both in terms of diet preference (Fig. 1B) and the effect on the reduction of leucine neosynthesis (Fig. 2) in the symbiotic pea aphids. Further studies are needed to explore the kinetic response of *B. aphidicola* to stresses which have an effect on symbiotic physiology, comparing young and adult aphids.

Although no correlation between tryptophan production and variations in the pTrp copy number has been observed (Birkle *et al*., 2002), our work shows a specific, albeit moderate, modulation of *B. aphidicola* pLeu plasmid copy number in response to variations in leucine concentration in the aphid diet starting 2 days after the beginning of the treatment (Fig. 4). The genes thought to be responsible for pLeu replication are the *rep* replicases, which are closely related to the *E. coli repA* gene, located in all the low copy number plasmids belonging to the IncFII group (Maas *et al*., 1991; de la Cueva-Méndez and Pimentel, 2007). Two genes of the pLeu plasmid in the Aphididae family code for replicases, an unusual feature for IncFII plasmids. The functional role of the presence of these two copies of *repA* in the *B. aphidicola* pLeu is completely unknown, although RepA1 and RepA2 have been annotated *in silico*, respectively, as the initiator and the regulator of pLeu replication (Pérez-Brocal *et al*., 2006). In our study, the two genes show strong induction and repression, respectively, following leucine starvation and excess, both of which precede dynamically the plasmid replication response suggesting that RepA1 and/or RepA2 levels could actually regulate pLeu amplification. Nevertheless, the amplitude of the transcriptional response for these two genes is not followed by a correspondingly strong regulation of the pLeu copy number variation. This may be explained by a partial loss in the capability of the Rep proteins to recognize the pLeu replication sequences in *B. aphidicola*. As intergenic regions are known to evolve very quickly, we can attribute this loss of replication capability to the degeneration of RepA targets. Indeed, ori seems to be the most variable pLeu intergenic region within pLeu between different *B. aphidicola* strains (data not shown) and, with the exception of the SIR1 and SIR2 sequences previously reported (Soler *et al*., 2000), we were not able to find, in the pea aphid leucine plasmid, any other canonical Rep targets (i.e. SIR3 and IR1/IR2), reported as being necessary for the IncFII plasmid Rep-dependent replication. These results do not exclude other currently unknown roles for the pLeu *rep* genes.

Finally, despite the important AT bias of the pLeu sequence, we were able to identify elements potentially involved in the transcriptional regulation of leucine biosynthetic genes in *B. aphidicola*. The presence of putative σ^70^ promoters, at the 5′ end regions of the *repA1*, *repA2* and *yqhA* genes, indicates that the transcription of each of these genes could be regulated independently. Also, only two σ^70^ putative promoter signals have been identified for *leuABCD* genes: one located upstream of the *leuA* gene and a second located in the *leuA* coding sequence, upstream of the *leuBCD* structure. Additionally, a 60 bp intergenic region (consisting of only 3 base pairs in *E. coli*) separates in the *B. aphidicola leuA* and *leuBCD* genes. RT-PCR experiments confirmed that *leuABCD* is present as a unique transcription unit in *B. aphidicola* in basal conditions (Fig. 5B). Nevertheless, our real-time qRT-PCR data show that, in conditions of leucine starvation, a different regulation seems to be acting on the *leuA* gene, when compared with the *leuBCD* genes (Fig. 3A). Based on the novel annotation of the *B. aphidicola* pLeu plasmid sequence presented here, this independent regulation could be explained by two different, but not mutually exclusive, mechanisms: (i) an independent transcription initiation of *leuA,* compared with the other genes of the operon, due to the presence of the internal putative promoter; and (ii) a difference in the stabilization properties of the *leuA* and *leuBCD* mRNAs thanks to the stem-loop structures present at the 5′ end of these genes. Without excluding other possible regulations coming for the pea aphid host, we hypothesize that the branch-point function of LeuA would have facilitated its selective individualization as a regulatory protein of the overall branched-chain amino acid superpathway.

## Experimental procedures

### Aphid rearing

A long-established parthenogenetic clone (LL01) of *Acyrthosiphon pisum* Harris, free of any of the five taxa of secondary endosymbionts identified to date, was maintained on broad beans (*Vicia faba* L. cv Aguadulce) at 21°C, with a 16-hour light photoperiod. Apterous viviparous adults, obtained from a synchronized cohort (Rahbé and Febvay, 1993), were allowed to lay progeny on young plants. All experiments were initiated by transferring neonate aphids (aged 0–6 h) from plants to artificial diets. Based on the nutritionally optimized Ap3 diet (Febvay *et al*., 1988), 10 diet formulations were used in this study, differing only in leucine concentration ( = conditions: from 0 to 90 mM). From our knowledge of the larval development of pea aphid on artificial diets and of the total amino acid content of whole aphid tissues (Febvay *et al*., 1988), 20 mM leucine was taken as the reference dietary concentration for the pea aphid.

### Growth assays and diet choice experiments

Growth assays were performed, as previously described (Rahbé and Febvay, 1993), using 54 insects per condition and scoring individual aphid weights at the end of a standard nymphal period (7 days, 21°C). For the aphid diet choice experiments, neonate *A. pisum* larvae were transferred onto artificial diets enclosed in sterile parafilm sachets and reared there throughout the larval developmental period. Choice tests were run and analysed, as described before (Sauvion *et al*., 2004), enclosing six neonate first instar aphid larvae in a dual choice cage with diets from either a control (20 mM leucine) or a test condition (0–90 mM leucine). Cages (*n* = 24) were left for aphid choice in a dark cabinet and aphid positions on control versus test diet were scored after 8 h. A Diet Preference Index [DPIx = (*T* − *C*)/(*T* + *C*) with *T* = aphids recorded on test diet; *C* = aphids recorded on control diet] was used to score the attractive effect of leucine, comparing complete avoidance (−1) to complete acceptance (+1) for the proposed diets, and significant differences were tested by a Wilcoxon non-parametric signed rank test.

### Amino acid neosynthesis quantification

The amino acid neosyntheses in symbiotic aphids, reared on artificial diets containing different concentrations of leucine, were measured as previously described (Rahbé*et al*., 2002). Neonate larvae collected from plants were deposited and reared on artificial diets with a leucine concentration ranging from 0 to 80 mM and containing U-^14^C sucrose (22.9 GBq mmol^−1^, Isotopchim, Ganagobie-Peyruis, France) at about 1 MBq ml^−1^. The initial radioactivity of the diet was counted in triplicate on 10 µl samples, after addition of 4.5 ml of Ultima Gold scintillation fluid (Packard Instrument SA, Rungis, France), using a Packard Tri-Carb 460C Liquid Scintillator System with a preset ^14^C window. After 7 days of treatment, five aphids (fourth instar) from each experimental condition were individually weighed and hydrolysed for 24 h, under nitrogen, in HCl vapour at 110°C and in the presence of 2-β-mercaptoethanol to preserve the sulphur amino acids, using the Pico-Tag work station (Waters, St Quentin-Les-Yvelines, France). After the addition of 50 nmol of glucosaminic acid (internal standard), the samples were dried under vacuum conditions and collected in 150 µl of 50 mM lithium citrate buffer, pH = 2.2. Triplicate aliquots of 10 µl were used for liquid scintillation counting as above, while 50 µl was used for amino acid analysis. In this case, the automatic amino acid analyser was coupled with an on-line radioactivity flow detector (Flo-one/Beta A500, Packard), which allowed for the quantification of radiolabelled compounds as described before (Febvay *et al*., 1999). The radioactivity recovered for each amino acid was then computed as sucrose nmol equivalents and expressed per mg of aphid fresh weight.

### Design of primer sets

All the primers (Table S2) used in this work (for real-time quantitative PCR and for the experimental validation of the *leuABCD* operon) were designed using the oligo 7 software (Rychlik, 2007). The chromosomal and plasmidic gene sequences were retrieved, respectively, from the *B. aphidicola* genome sequence data (Shigenobu *et al*., 2000) (NC 002528.1; GI: 15616630 in GenBank) and the pLeu sequence previously published (Silva *et al*., 1998) (GenBank ID: AJ006878).

### Differential gene expression analysis by real-time quantitative RT-PCR

Neonate larvae, collected from plants, were deposited and reared on artificial diets containing 0, 20 or 60 mM leucine. In order to acquire a dynamic response of *B. aphidicola*, aphids were maintained on the artificial diets for 12 h, 1, 2, 3 or 7 days ( = five time points). Three groups of 333 aphids ( = three independent biological replicates) were used for each of the three tested conditions and at each of the five time points. For each of these samples, total RNA was extracted independently and 1 µg of it was retro-transcribed and then used for quantitative PCR reactions. Briefly, symbiotic bacteria were purified from aphids, as described by Charles and Ishikawa (1999), and the protocol used for RNA extraction was the same as that previously described by Calevro and colleagues (2004). Total RNA was then purified using the TRIzol method and possible gDNA contaminants were removed using DNase RQ1 RNase-free (Promega, Madison, WI, USA). Total RNA was subsequently purified on an RNeasy column (Qiagen, Hilden, Germany). The quality of extracted RNA was verified on agarose gels in denaturing conditions and the concentrations were measured using a NanoDrop ND-1000 spectrophotometer (NanoDrop Technologies, Wilmington, DE, USA).

For the real-time quantitative RT-PCR experiments, we measured the transcriptomic response of the seven pLeu genes (*leuABCD*, *repA1*, *repA2*, *yqhA*). In addition to these genes, we also included in our analysis the chromosomal genes *ilvI* and *ilvH*, which are of special interest in leucine biosynthesis as they encode, respectively, the large and the small subunit of the acetolactate synthase enzyme, able to catalyse the first steps of the branched-chain amino acid superpathway (Fig. S1). These two genes are known to play a key role in the regulation of leucine biosynthesis in free-living bacteria (Umbarger, 1987).

Three genes have been initially chosen for data normalization: *atpA*, *rplX* and *rpmC*. These genes have been selected because they have been shown to be invariant genes in microarray datasets in several tested conditions in *B. aphidicola* (Moran *et al*., 2005; Charles *et al*., 2006; Reymond *et al*., 2006). Among these three genes, the best candidates for data normalization were tested using the *BestKeeper* software tool (Pfaffl *et al*., 2004). Only *atpA* and *rplX* were retained as normalization genes as they met the criteria imposed by the BestKeeper analysis: standard deviation ≤ 1 CP between the three tested conditions for each experimental time point (Table S3). To prepare the real-time PCR assay, total RNA was reversed transcribed using the SuperScript III First-Strand Synthesis System for RT-PCR (Invitrogen, Paisley, UK) in the presence of random hexamers. The quantification of mRNA levels by real-time PCR was performed in 96-well plates with a LightCycler 480 instrument (Roche diagnostics, Meylan, France). The measurement was performed in a 10 µl final volume of reaction mixture (containing 2.5 µl of 1/5 diluted cDNA template), prepared following the instructions of the Light Cycler 480 SYBR Green I Kit (Roche diagnostics) with the primer set at a final concentration of 0.5 µM. An internal standard curve was generated for each tested gene using serial dilutions (from 2000 to 0.02 fg µl^−1^) of purified PCR products amplified from genomic DNA. PCR reactions were initiated by the activation of Taq DNA polymerase at 95°C for 5 min, followed by 45 three-step amplification cycles consisting of 15 s denaturation at 95°C, 15 s annealing at 47°C and 15 s extension at 72°C. The fluorescent signal was measured at the end of each extension step. After the amplification, a dissociation stage was run to generate a melting curve for verification of amplification product specificity, which was also confirmed on agarose gels. The crossing point (CP) was determined by the ‘Second derivative maximum method’ in the LightCycler 480 Software release 1.5.0. The overall transcriptional response of *B. aphidicola* (CP values) before data normalization is shown in Table S4.

The gene expression levels of all the target genes were calculated and normalized using the *REST* software tool (Pfaffl *et al*., 2002). The relative expression ratio of each target gene was calculated by comparing the tested condition versus the control condition and relative to the normalization genes. More precisely, this ratio (*R*) was calculated taking into account the real-time PCR efficiency of each gene (*E*) and the crossing point difference (ΔCP) of a test condition (0 or 60 mM leucine), as compared with the reference condition (20 mM leucine), and expressed in comparison to the normalization genes (*atpA* and *rplX*) using the following model (Pfaffl, 2001):

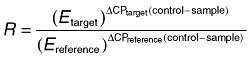


### *B. aphidicola* pLeu plasmid copy number quantification

As for gene expression quantification, neonate larvae collected from plants were deposited on three artificial diets (with 0, 20 or 60 mM leucine) and reared there for 12 h, 1, 2, 3 or 7 days. Three groups of 333 individuals, corresponding to three independent biological replicates, were used for each condition and at each time point. After purification of *B. aphidicola* from aphids, total DNA was extracted using the QIAamp DNA Mini Kit (Qiagen), following the procedure adapted to bacterial cells as described in the manufacturer's instructions. The optional treatment, of 10 min at 70°C with RNase A (Qiagen), was performed during the extraction procedure. The quality of extracted DNA was verified on 1.5% agarose gels and the concentrations were measured using a NanoDrop ND-1000 spectrophotometer. The template DNA extracted from *B. aphidicola* was normalized to 2.5 ng µl^−1^ with ultrapure water for real-time quantitative PCR experiments.

The pLeu plasmid copy number was quantified relative to a single chromosomal gene copy, as described by Plague *et al*. (2003). To take into account a possible bias due to plasmid replication, we measured the pLeu copy number using three genes, uniformly distributed along the plasmid: *leuA*, *leuC* and *yqhA*. The chromosomal *atpA* gene was used for normalization in this experiment. For the three independent experiments, the quantification of each gene copy number was performed by real-time quantitative PCR in 96-well plates, in a 10 µl final volume of reaction mixture prepared following the instructions of the Light Cycler 480 SYBR Green I Kit, with 2.5 µl template DNA and the primer sets at a final concentration of 0.5 µM. The internal standard curve and the PCR reaction were performed as described above for the gene expression analysis.

The gene copy number was calculated using the following equation (Whelan *et al*., 2003):

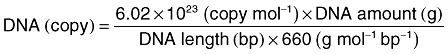
for each of the three target genes independently and then normalized by the *atpA* response.

Mean comparisons were then performed to verify the homogeneity of the three gene responses in each experimental group, before averaging the data.

### Prediction of regulatory regions in the *B. aphidicola* pLeu plasmid

The pLeu plasmid was re-annotated from EMBL/GB accession AJ006878.2 (GI:5912533, 7805 bp), which was sequenced from the *A. pisum* LL01 clone (Silva *et al*., 1998), using the MacVector software (MacVector, Cary, NC, USA) and some routines of the genomic software Iogma (Genostar, Paris, France). Re-annotation included identification of: (i) ribosome binding site signals upstream of the plasmid coding sequences (cds) via RBS finder (Suzek *et al*., 2001); (ii) rho-independent intrinsic terminators and stem-loops via Termit in Iogma (T. Vermat and Y. Vandenbrouck, unpublished) and TransTermHP (Kingsford *et al*., 2007); (iii) σ^70^ putative promoters via the BPROM promoter prediction tool (http://linux1.softberry.com/berry.phtml?topic = gfindb); and (iv) miscellaneous repeated sequences identified with the Pustell matrix analysis within MacVector. Finally, to test our promoter predictions, a comparative evolutionary analysis was run on plasmids from 10 aphid species within the Aphididae family. Because most AT-rich intergenic regions could not be aligned, with the exception of within-tribe comparisons in certain regions as has been previously reported (Silva *et al*., 1998), we used BPROM predictions on all species to look for signal conservation.

### Experimental validation of the leuABCD operon in *B. aphidicola*

The existence of the putative *leuABCD* operon was tested by RT-PCR following the procedure, developed on *Streptomyces coelicolor,* to detect the operon organization of bacterial genes (Charaniya *et al*., 2007) and recently adapted to the *B. aphidicola* genome (Brinza *et al*., 2010).

Total RNA from *B. aphidicola* was treated with DNase (2 units per 10 µg of RNA) for 1 h at 37°C, following the instructions of the Turbo DNA-free kit (Ambion Austin, TX, USA). Total RNA was subsequently purified on an RNeasy column (Qiagen). The quality of extracted RNA was verified on agarose gels in denaturing conditions and the concentrations were measured using a NanoDrop ND-1000 spectrophotometer.

A reverse transcription reaction was then performed, from 1 µg of total RNA, using the SuperScript III First-Strand Synthesis System for RT-PCR (Invitrogen). Samples were first incubated for 5 min at 65°C, prior to being cooled at 4°C for 1 min. Ten microlitres of reverse transcription mixture was added to the sample and a series of incubations was performed: 10 min at 25°C, 50 min at 50°C, 5 min at 85°C, and at least 2 min at 4°C. Finally, the initial RNA matrix was degraded for 20 min at 37°C, after the addition of 1 µl of RNase H.

The PCR reaction was carried out on 2 µl of reverse transcription product using the AccuPrime *Taq* DNA Polymerase High Fidelity Kit (Invitrogen), adapted to amplify DNA fragments of up to 20 kb and according to the following protocol: activation of Taq DNA polymerase at 95°C for 30 s, followed by 35 three-step amplification cycles consisting of 30 s denaturation at 95°C, 30 s annealing at 50.8°C, and 4 min of extension at 68°C.

### Statistical analysis

Statistical analyses of the data and the mean comparisons (anova*F*-test, Dunett's test, Tukey–Kramer HSD test, Student–Newman–Keuls test and Wilcoxon non-parametric signed rank test) were performed using JMP 5.0.1.2 software (SAS Institute, Cary, NC, USA).

## References

[b1] Baumann P (2005). Biology of bacteriocyte-associated endosymbionts of plant sap-sucking insects. Annu Rev Microbiol.

[b2] Birkle LM, Minto LB, Douglas AE (2002). Relating genotype and phenotype for tryptophan synthesis in an aphid-bacterial symbiosis. Physiol Entomol.

[b3] Bracho AM, Martínez-Torres D, Moya A, Latorre A (1994). Discovery and molecular characterization of a plasmid localized in *Buchnera* sp. bacterial endosymbiont of the aphid *Rhopalosiphum padi*. J Mol Evol.

[b4] Brinza L, Viñuelas J, Cottret L, Calevro F, Rahbé Y, Febvay G (2009). Systemic analysis of the symbiotic function of *Buchnera aphidicola*, the primary endosymbiont of the pea aphid *Acyrthosiphon pisum*. C R Biol.

[b5] Brinza L, Calevro F, Duport G, Gaget K, Gautier C, Charles H (2010). Structure and dynamics of the operon map of *Buchnera aphidicola* sp. strain APS. BMC Genomics.

[b6] Calevro F, Charles H, Reymond N, Dugas V, Cloarec JP, Bernillon J (2004). Assessment of 35mer amino-modified oligonucleotide based microarray with bacterial samples. J Microbiol Methods.

[b7] Chang DE, Smalley DJ, Conway T (2002). Gene expression profiling of *Escherichia coli* growth transitions: an expanded stringent response model. Mol Microbiol.

[b8] Charaniya S, Mehra S, Lian W, Jayapal KP, Karypis G, Hu WS (2007). Transcriptome dynamics-based operon prediction and verification in *Streptomyces coelicolor*. Nucleic Acids Res.

[b9] Charles H, Ishikawa H (1999). Physical and genetical map of the genome of *Buchnera*, the primary endosymbiont of the pea aphid *Acyrthosiphon pisum*. J Mol Evol.

[b10] Charles H, Calevro F, Viñuelas J, Fayard JM, Rahbé Y (2006). Codon usage bias and tRNA over-expression in *Buchnera aphidicola* after aromatic amino acid nutritional stress on its host *Acyrthosiphon pisum*. Nucleic Acids Res.

[b11] de la Cueva-Méndez G, Pimentel B (2007). Gene and cell survival: lessons from prokaryotic plasmid R1. EMBO Rep.

[b12] Daley DO, Rapp M, Granseth E, Melen K, Drew D, von Heijne G (2005). Global topology analysis of the *Escherichia coli* inner membrane proteome. Science.

[b13] Douglas AE (1988). Sulphate utilization in an aphid symbiosis. Insect Biochem.

[b14] Douglas AE, Prosser WA (1992). Synthesis of the essential amino acid tryptophan in the pea aphid (*Acyrthosiphon pisum*) symbiosis. J Insect Physiol.

[b15] Douglas AE, Minto LB, Wilkinson TL (2001). Quantifying nutrient production by the microbial symbionts in an aphid. J Exp Biol.

[b16] Durfee T, Hansen AM, Zhi H, Blattner FR, Jin DJ (2008). Transcription profiling of the stringent response in *Escherichia coli*. J Bacteriol.

[b17] Febvay G, Delobel B, Rahbé Y (1988). Influence of the amino acid balance on the improvement of an artificial diet for a biotype of *Acyrthosiphon pisum* (Homoptera: Aphididae). Can J Zool.

[b18] Febvay G, Liadouze I, Guillaud J, Bonnot G (1995). Analysis of energetic aminoacid metabolism in *Acyrthosiphon pisum*: a multidimensional approach to amino acid metabolism in aphids. Arch Insect Biochem Physiol.

[b19] Febvay G, Rahbé Y, Rynkiewicz M, Guillaud J, Bonnot G (1999). Fate of dietary sucrose and neosynthesis of amino acids in the pea aphid, *Acyrthosiphon pisum*, reared on different diets. J Exp Biol.

[b20] Gattolin S, Newbury HJ, Bale JS, Tseng HM, Barrett DA, Pritchard J (2008). A diurnal component to the variation in sieve tube amino acid content in wheat. Plant Physiol.

[b21] Gholami M, Coombe BG, Robinson SR (2004). Grapevine phloem sap analysis: 1-sucrose, amino acids, potassium concentrations, seasonal and diurnal patterns. Acta Hort (ISHS).

[b22] Gil R, Sabater-Muñoz B, Pérez-Brocal V, Silva FJ, Latorre A (2006). Plasmids in the aphid endosymbiont *Buchnera aphidicola* with the smallest genomes. A puzzling evolutionary story. Gene.

[b23] van Ham RCH, Moya A, Latorre A (1997). Putative evolutionary origin of plasmids carrying the genes involved in leucine biosynthesis in *Buchnera aphidicola* (endosymbiont of aphids). J Bacteriol.

[b24] van Ham RCH, Kamerbeek J, Palacios C, Rausell C, Abascal F, Bastolla U (2003). Reductive genome evolution in *Buchnera aphidicola*. Proc Natl Acad Sci USA.

[b25] International Aphid Genomics Consortium (2010). Genome sequence of the pea aphid *Acyrthosiphon pisum*. PLoS Biol.

[b26] Khodursky AB, Peter BJ, Cozzarelli NR, Botstein D, Brown PO, Yanofsky C (2000). DNA microarray analysis of gene expression in response to physiological and genetic changes that affect tryptophan metabolism in *Escherichia coli*. Proc Natl Acad Sci USA.

[b27] Kingsford CL, Ayanbule K, Salzberg SL (2007). Rapid, accurate, computational discovery of Rho-independent transcription terminators illuminates their relationship to DNA uptake. Genome Biol.

[b28] Latorre A, Gil R, Silva FJ, Moya A (2005). Chromosomal stasis versus plasmid plasticity in aphid endosymbiont *Buchnera aphidicola*. Heredity.

[b29] Liadouze I, Febvay G, Guillaud J, Bonnot G (1995). Effect of diet on the free amino acid pools of symbiotic and aposymbiotic pea aphids, *Acyrthosiphon pisum*. J Insect Physiol.

[b30] Maas R, Oppenheim J, Saadi S, Fuchs T, Maas WK (1991). Isolation and properties of the RepA1 protein of the IncFII replicon, RepFIC. Mol Microbiol.

[b31] Macdonald SJ, Thomas GH, Douglas AE (2011). Genetic and metabolic determinants of nutritional phenotype in an insect-bacterial symbiosis. Mol Ecol.

[b32] Moran NA, Degnan PH (2006). Functional genomics of *Buchnera* and the ecology of aphid hosts. Mol Ecol.

[b33] Moran NA, Munson MA, Baumann P, Ishikawa H (1993). A molecular clock in endosymbiotic bacteria is calibrated using the insect hosts. Proc R Soc B.

[b34] Moran NA, Plague GR, Sandstrom JP, Wilcox JL (2003). A genomic perspective on nutrient provisioning by bacterial symbionts of insects. Proc Natl Acad Sci USA.

[b35] Moran NA, Dunbar HE, Wilcox JL (2005). Regulation of transcription in a reduced bacterial genome: nutrient-provisioning genes of the obligate symbiont *Buchnera aphidicola*. J Bacteriol.

[b36] Moran NA, McLaughlin HJ, Sorek R (2009). The dynamics and time scale of ongoing genomic erosion in symbiotic bacteria. Science.

[b37] Park JH, Lee KH, Kim TY, Lee SY (2007). Metabolic engineering of *Escherichia coli* for the production of L-valine based on transcriptome analysis and *in silico* gene knockout simulation. Proc Natl Acad Sci USA.

[b38] Pate J, Shedley E, Arthur D, Adams M (1998). Spatial and temporal variations in phloem sap composition of plantation-grown *Eucalyptus globulus*. Oecologia.

[b39] Pérez-Brocal V, Gil R, Ramos S, Lamelas A, Postigo M, Michelena JM (2006). A small microbial genome: the end of a long symbiotic relationship?. Science.

[b40] Pfaffl MW (2001). A new mathematical model for relative quantification in real-time RT-PCR. Nucleic Acids Res.

[b41] Pfaffl MW, Horgan GW, Dempfle L (2002). Relative expression software tool (REST) for group-wise comparison and statistical analysis of relative expression results in real-time PCR. Nucleic Acids Res.

[b42] Pfaffl MW, Tichopad A, Prgomet C, Neuvians TP (2004). Determination of stable housekeeping genes, differentially regulated target genes and sample integrity: BestKeeper–Excel-based tool using pair-wise correlations. Biotechnol Lett.

[b43] Plague GR, Dale C, Moran NA (2003). Low and homogeneous copy number of plasmid-borne symbiont genes affecting host nutrition in *Buchnera aphidicola* of the aphid *Uroleucon ambrosiae*. Mol Ecol.

[b44] Rahbé Y, Febvay G (1993). Protein toxicity to aphids – an *in vitro* test on *Acyrthosiphon pisum*. Entomol Exp Appl.

[b45] Rahbé Y, Digilio MC, Febvay G, Guillaud J, Fanti P, Pennacchio F (2002). Metabolic and symbiotic interactions in amino acid pools of the pea aphid *Acyrthosiphon pisum* parasitized by the braconid *Aphidius ervi*. J Insect Physiol.

[b46] Reymond N, Calevro F, Viñuelas J, Morin N, Rahbé Y, Febvay G (2006). Different levels of transcriptional regulation due to trophic constraints in the reduced genome of *Buchnera aphidicola* APS. Appl Environ Microbiol.

[b47] Rychlik W (2007). OLIGO 7 primer analysis software. Methods Mol Biol.

[b48] Sabater-Muñoz B, van Ham RC, Moya A, Silva FJ, Latorre A (2004). Evolution of the leucine gene cluster in *Buchnera aphidicola*: insights from chromosomal versions of the cluster. J Bacteriol.

[b49] Sasaki T, Ishikawa H (1995). Production of essential amino acids from glutamate by mycetocyte symbionts of the pea aphid, *Acyrthosiphon pisum*. J Insect Physiol.

[b50] Sauvion N, Charles H, Febvay G, Rahbé Y (2004). Effects of the jackbean lectin (ConA) on the feeding behaviour and kinetics of intoxication of the pea aphid, *Acyrthosiphon pisum* (Harris). Entomol Exp Appl.

[b51] Sharkey PJ, Pate JS (1976). Translocation from leaves to fruits of a legume, studied by a phloem bleeding technique: diurnal changes and effects of continuous darkness. Planta.

[b52] Shigenobu S, Watanabe H, Hattori M, Sakaki Y, Ishikawa H (2000). Genome sequence of the endocellular bacterial symbiont of aphids *Buchnera* sp. APS. Nature.

[b53] Silva FJ, van Ham RCH, Sabater B, Latorre A (1998). Structure and evolution of the leucine plasmids carried by the endosymbiont (*Buchnera aphidicola*) from aphids of the family Aphididae. FEMS Microbiol Lett.

[b54] Smith JAC, Miburn JA (1980). Phloem transport, solute flux and the kinetics of sap exudation in *Ricinus communis* L. Planta.

[b55] Soler T, Latorre A, Sabater B, Silva FJ (2000). Molecular characterization of the leucine plasmid from *Buchnera aphidicola*, primary endosymbiont of the aphid *Acyrthosiphon pisum*. Curr Microbiol.

[b56] Suzek BE, Ermolaeva MD, Schreiber M, Salzberg SL (2001). A probabilistic method for identifying start codons in bacterial genomes. Bioinformatics.

[b57] Tamas I, Klasson L, Canback B, Naslund AK, Eriksson AS, Wernegreen JJ (2002). 50 million years of genomic stasis in endosymbiotic bacteria. Science.

[b58] Thao ML, Baumann L, Baumann P, Moran NA (1998). Endosymbionts (*Buchnera*) from the aphids *Schizaphis graminum* and *Diuraphis noxia* have different copy numbers of the plasmid containing the leucine biosynthetic genes. Curr Microbiol.

[b59] Thomas GH, Zucker J, Macdonald SJ, Sorokin A, Goryanin I, Douglas AE (2009). A fragile metabolic network adapted for cooperation in the symbiotic bacterium *Buchnera aphidicola*. BMC Syst Biol.

[b60] Umbarger HE, Neidhardt JLIFC, Low KL, Magasanik B, Schaechter M, Umbarger HE (1987). Biosynthesis of the branched chain amino acids. *Escherichia coli* and *Salmonella typhimurium:* Cellular and Molecular Biology.

[b61] Viñuelas J, Calevro F, Remond D, Bernillon J, Rahbé Y, Febvay G (2007). Conservation of the links between gene transcription and chromosomal organization in the highly reduced genome of *Buchnera aphidicola*. BMC Genomics.

[b62] Von Dohlen CD, Moran NA (2000). Molecular data support a rapid radiation of aphids in the Cretaceous and multiple origins of host alternation. Biol J Linn Soc.

[b63] Wernegreen JJ (2002). Genome evolution in bacterial endosymbionts of insects. Nat Rev Genet.

[b64] Whelan JA, Russell NB, Whelan MA (2003). A method for the absolute quantification of cDNA using real-time PCR. J Immunol Methods.

[b65] Wilson AC, Ashton PD, Calevro F, Charles H, Colella S, Febvay G (2010). Genomic insight into the amino acid relations of the pea aphid, *Acyrthosiphon pisum*, with its symbiotic bacterium *Buchnera aphidicola*. Insect Mol Biol.

[b66] Winter H, Lohaus G, Heldt HW (1992). Phloem transport of amino acids in relation to their cytosolic levels in barley leaves. Plant Physiol.

