# Supplementary material for: Multimodal dynamic response of the Buchnera aphidicola pLeu plasmid to variations in leucine demand of its host, the pea aphid Acyrthosiphon pisum
Source: Mol Microbiol. 2011 Jul 28;81(5):1271–85. doi: 10.1111/j.1365-2958.2011.07760.x (PMC3229713; doi:10.1111/j.1365-2958.2011.07760.x)
Supplement: Supplementary file 1 [file mmi0081-1271-SD1.pdf]

Multimodal dynamic response of the *Buchnera aphidicola* pLeu plasmid to variations in leucine demand of its host, the pea aphid *Acyrthosiphon pisum*

José Viñuelas[†], Gérard Febvay, Gabrielle Duport, Stefano Colella, Jean-Michel Fayard,

Hubert Charles, Yvan Rahbé and Federica Calevro*

UMR203 BF2I, Biologie Fonctionnelle Insectes et Interactions, INSA-Lyon, INRA,

Université de Lyon, Bât. Louis Pasteur, 20 av. Albert Einstein, F-69621 Villeurbanne, France.

*For correspondence.
E-mail federica.calevro@insa-lyon.fr
Tel. (+33) 472 43 79 88
Fax (+33) 472 43 85 34

† Present address:
Université Lyon 1, FST, Lyon, F-69003, France ; CNRS, UMR 5534, CGphiMC, F-69622 Villeurbanne, France.

Supporting Information

Figure S1. Pathways for the biosynthesis of isoleucine, leucine and valine.
The pathways were modified from http://acypicyc.cycadsys.org/ and completed with data from Wilson and colleagues (Wilson *et al.,* 2010). The enzymes encoded by *B. aphidicola* but absent in the pea aphid, and *vice versa*, are specified in blue and in green, respectively.

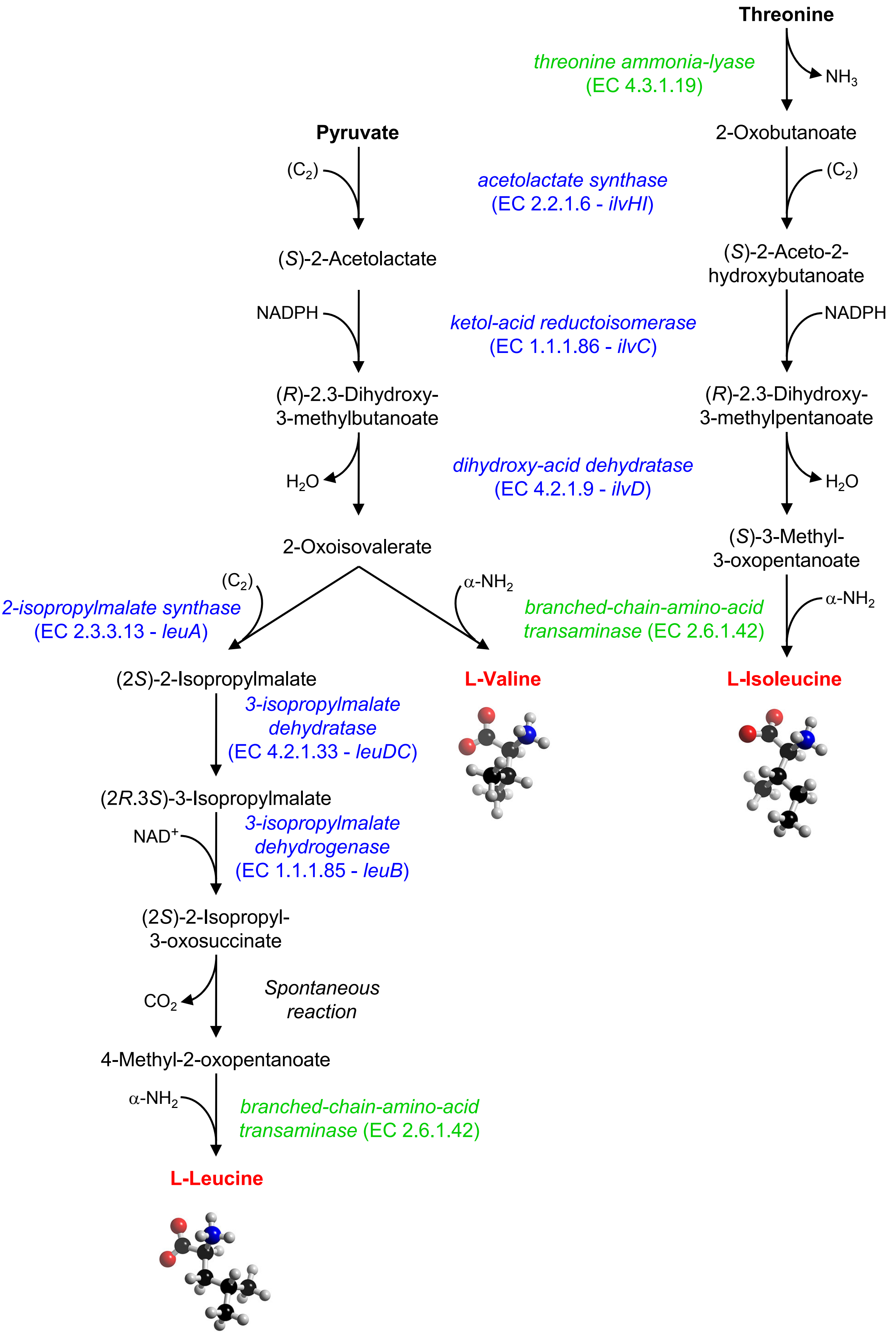

Figure S2. Features map of the reannotated pLeu plasmid in *B. aphidicola* from *A. pisum.*

Stem-loop structures are shown in green (SL), promoter sequences in brown (Prom.), rho-independent terminators in red (Term.) and ribosomal binding sites in blue (RBS).

```
       Ori                                    SL1
       ~                ~~~~~~~~~~~~~~~~~~~~~~~~~~~~~~~~~~~~~~~~~~~~~~~~~~~~
    1  ATGTTATTAT TTTTTTAAAA AAATAGCGAA GTTTTTAGAA ATAAAAAACT TCGCTATTTT TTTTTAATTT AACAAGTATG
                                ~~~~~~~~~~~~~~~~~~~~~~~~~~~~~~~~~~~~
                                             Term. 1
   81  TTTTTATTTA TCTTTTTATA TAAGTTTTAA GAATGTTATT TTGCATTTAT TTATATAAGA AGCATTTATT AAAAAAAACA
                                              SL2
                    ~~~~~~~~~~~~~~~~~~~~~~~~~~~~~~~~~~~~~~~~~~~~~~~~~~~~~~~~~~~~~
  161  GTATGCATCT ATTTATTTTT TCAATAAAAA AATGTGATTC TAATATTTTT TTTTATTGAA AAAATAAAAT AAGATGTTTT

  241  ATGATAAAAA AAAAGGGGAG AGATAATTTT ACAGTGGATT TAAGAAACAA AAAATATGCG AATAAACGCA TGCCTTTAAG
                                                                     ~~~~~~~~~~~~~~~~~~
                                                                         Term. 2
  321  AAAGAATAAA TTTAAATAAA AATTTTAATT ATCAGGATAA GTGTTTGTAG CTATCTTGCG TAAATAATAA TAGCTTATAT
                                 ~~~~~~~~~~~~~~~~~~~~~~~~~~~~~~~~~~~~~~~~~~~~~~~~~~~~~~~~~~~~
                                                    repA1 (345 - 1196)
  401  TAACTTGTTT CTTTAATCCA TTAGGCCCTA ATTTAGTTAA TTCAGTAATA GAGTATTTGG CTACTAACGC TCGAAGAATA
       ~~~~~~~~~~~~~~~~~~~~~~~~~~~~~~~~~~~~~~~~~~~~~~~~~~~~~~~~~~~~~~~~~~~~~~~~~~~~~~~~~~~~~~~

  481  GTTTGTCTGG CTTCTTTCTC ATCTAATGAA ATCAAGCGTT GTGCATTTCT CTTTTTTTTA TAAAAAGCAT GCTTAGAAAT
       ~~~~~~~~~~~~~~~~~~~~~~~~~~~~~~~~~~~~~~~~~~~~~~~~~~~~~~~~~~~~~~~~~~~~~~~~~~~~~~~~~~~~~~~

  561  TCTATATTGA AAAATATTTT TAATTTGAGT ATCTTTAGAA CGTCTTTTAG CATCAATCAT GGTTATTGGT TTAAAACCTT
       ~~~~~~~~~~~~~~~~~~~~~~~~~~~~~~~~~~~~~~~~~~~~~~~~~~~~~~~~~~~~~~~~~~~~~~~~~~~~~~~~~~~~~~~

  641  TACTAATGAG ATTTTTATTG ATCCACCCTA ATTGTTGTTT CTTAGCATCC ATTAATTTTT TTTCAGAAAC ACCAAAGAGC
       ~~~~~~~~~~~~~~~~~~~~~~~~~~~~~~~~~~~~~~~~~~~~~~~~~~~~~~~~~~~~~~~~~~~~~~~~~~~~~~~~~~~~~~~
                                                Term. 3
                                         ~~~~~~~~~~~~~~~~~~~~~~
  721  ATAAAAAACA ATGGAGTTAA TGTGATCATT TTGGGCATAT AATTGCCTAA TATTTTATCC CATGTTTTTT TGCAAGTAAC
       ~~~~~~~~~~~~~~~~~~~~~~~~~~~~~~~~~~~~~~~~~~~~~~~~~~~~~~~~~~~~~~~~~~~~~~~~~~~~~~~~~~~~~~~

  801  AAAACCCATA GGTTCCATAA AATTAGTAAT TAATCTAGAA GCACGAGTAA TAGATTTATT ACCTGCTTTA GACAAGGTAG
       ~~~~~~~~~~~~~~~~~~~~~~~~~~~~~~~~~~~~~~~~~~~~~~~~~~~~~~~~~~~~~~~~~~~~~~~~~~~~~~~~~~~~~~~

  881  ACAAACCACA TTCATCAGAT AATTGTTCAA CTGATGCTTG AACCAAATCA GAAGAAATAT TAAAATGGTA AAGCATAGCT
       ~~~~~~~~~~~~~~~~~~~~~~~~~~~~~~~~~~~~~~~~~~~~~~~~~~~~~~~~~~~~~~~~~~~~~~~~~~~~~~~~~~~~~~~

  961  AAAACCATGG CTCTCATAGC ACATGCTCGG TGTTCATTTA ATCTTCTAAA TCTTTTTACG GGAAAACCAA TTTTAATATT
       ~~~~~~~~~~~~~~~~~~~~~~~~~~~~~~~~~~~~~~~~~~~~~~~~~~~~~~~~~~~~~~~~~~~~~~~~~~~~~~~~~~~~~~~

 1041  TTTGATCTGT AAAATATAAT TTAGTTCGCA TCGAGCCACA TCTATTTCTG ATGCTCTTTT CATTGCATAA CAAATAAAAG
       ~~~~~~~~~~~~~~~~~~~~~~~~~~~~~~~~~~~~~~~~~~~~~~~~~~~~~~~~~~~~~~~~~~~~~~~~~~~~~~~~~~~~~~~

 1121  ATGGCCTACG TTTATTATTT TTAGGTGGTG TAAAAAAATGG ATTGGGATTA TTTATATAGC ATTTTCTTAT TATCATAACA
       ~~~~~~~~~~~~~~~~~~~~~~~~~~~~~~~~~~~~~~~~~~~~~~~~~~~~~~~~~~~~~~~~~~~~~~~~~~~~~~~~~~~
                                                 SL3
                                     ~~~~~~~~~~~~~~~~~~~~~~~~~~~~
 1201  TGATACTTAA AATAGTAAGT GGGTTCATTT TTATATATTT CAAATATATA AAATTTTATT TTTATTCTTT CAATAATTAA
                                                       ~~~~~~~~~~~~~~~~~~~~~~~~~~~~~~~~~~~~~~~
                              Prom. 2                                     Prom. 1
                   ~~~~~~~~~~~~~~~~~~~~~~~~~~~~~~~~~~~~~~~
 1281  ATTTTTTTAT ATTTAAGAAT ATATACTTTA CTTTTATTTT TTTTTATGAA TAAATATAAT TTATAAAAAA TAAATATATT
       ~
 1361  TTTAACATAC ATTTTGATTA TTTTCAACGA TAACTTACTA AAACTTAAAA GTTTTATCTT AACTTTCTTA AATTAAATAA
                                                              RBS 1
                                                              ~~~~~                  ~~~~~~~~~~
 1441  AATATATTTA TAAATTCAAT CTATATCAAA AACAATATTT TTTATTAACA ATTACTATTC AGGTTAAAAA AATGGAAAAA
                                      yqhA (1512 - 2015)
       ~~~~~~~~~~~~~~~~~~~~~~~~~~~~~~~~~~~~~~~~~~~~~~~~~~~~~~~~~~~~~~~~~~~~~~~~~~~~~~~~~~~~~~~
 1521  ATTATTGAAA AAAGTATATA TGCCTCTCGT TGGTTAATGT TTCCTGTATA TGTAGGTTTA TCGTTTGGTT TTATTTTATT

       ~~~~~~~~~~~~~~~~~~~~~~~~~~~~~~~~~~~~~~~~~~~~~~~~~~~~~~~~~~~~~~~~~~~~~~~~~~~~~~~~~~~~~~~
 1601  AACATTGAAA TTTTTCCAAC AAATTGTATT TATCATGCCA GATATTGTGG CTATGTCAGA ATCAGGTTTA GTGTTAGTTG

       ~~~~~~~~~~~~~~~~~~~~~~~~~~~~~~~~~~~~~~~~~~~~~~~~~~~~~~~~~~~~~~~~~~~~~~~~~~~~~~~~~~~~~~~
```

```
1681  TTTTGTCATT AATTGATATT GCATTAGTAG GTGGTCTATT AGTTATGGTT ATGTTTTCTG GATATGAAAA TTTTATTTCT

      ~~~~~~~~~~~~~~~~~~~~~~~~~~~~~~~~~~~~~~~~~~~~~~~~~~~~~~~~~~~~~~~~~~~~~~~~~~~~~~~~~~~~~~~
1761  AAAATGGATA TACAAGATAA TCAGAAAAGA TTAGGGTGGA TGGGAACCAT GGATGTTAAT TCAATAAAAA ACAAAGTAGC

      ~~~~~~~~~~~~~~~~~~~~~~~~~~~~~~~~~~~~~~~~~~~~~~~~~~~~~~~~~~~~~~~~~~~~~~~~~~~~~~~~~~~~~~~
1841  ATCTTCAATA GTTGCCATAT CTTCTGTTCA TCTTTTACGT CTTTTTATGG AAGCAGAAAA AATATTAGAT GATAAAATTA

      ~~~~~~~~~~~~~~~~~~~~~~~~~~~~~~~~~~~~~~~~~~~~~~~~~~~~~~~~~~~~~~~~~~~~~~~~~~~~~~~~~~~~~~~
1921  TGTTATGTGT TATTATTCAT TTAACTTTTG TATTATCGGC TTTTGGAATG GCATATATTG ATAAAATGAG CAAAAAAAAA
                                                                      Prom. 3
      ~~~~~~~~~~~~~~~~                                          ~~~~~~~~~~~~~~~~~~~~~~~~~~~~~
2001  CATATCCTTC ATTAGTTTTT GTTTAAAAAA AATTTATTTT TTTTTATTAA TTTTTTATAT TTTTTTAAAA AAACTAATAT
                                                                                 RBS 2
      ~~~~~~~                                                                    ~~~~~    ~
2081  AAAAGTATAT TGTTCAATAT ATGATTTATC AAAAACTCTT CTTTTAAAAA AATATTTTAC TTTTAATTAA GTGTATTTAG
                                repA2 (2160 - 2912)
      ~~~~~~~~~~~~~~~~~~~~~~~~~~~~~~~~~~~~~~~~~~~~~~~~~~~~~~~~~~~~~~~~~~~~~~~~~~~~~~~~~~~~~~~
2161  TGCCTAGAAA AAATTATATA TATAATCTAA AACCATTTTT TAATCCATCT AAAAATGAAA GAAAAAAATC TACATTTATT

      ~~~~~~~~~~~~~~~~~~~~~~~~~~~~~~~~~~~~~~~~~~~~~~~~~~~~~~~~~~~~~~~~~~~~~~~~~~~~~~~~~~~~~~~
2241  TGTTATGCAA TGAAAAAAGT ATCAGAAATA GACGTTGCTA GAAGTCATTT AAATCGTGCA TTATTACCAA TAGATCCTAA

      ~~~~~~~~~~~~~~~~~~~~~~~~~~~~~~~~~~~~~~~~~~~~~~~~~~~~~~~~~~~~~~~~~~~~~~~~~~~~~~~~~~~~~~~
2321  AACTGGTAAT GTTCTGCCTC GATTCAGACG ATTAAATAAA CATCGAGCAT GTGCTATGAG AGCTATAGTA CCTGCTATGT

      ~~~~~~~~~~~~~~~~~~~~~~~~~~~~~~~~~~~~~~~~~~~~~~~~~~~~~~~~~~~~~~~~~~~~~~~~~~~~~~~~~~~~~~~
2401  TGTATTATTT TAATATTGAC TCTAAATTAG TCGAAGCTTC TATTGAAAAA TTAGCAGATG AATGTGGATT ATCTACTTTA
                                           Term. 4
                                      ~~~~~~~~~~~~~~~~~~~
      ~~~~~~~~~~~~~~~~~~~~~~~~~~~~~~~~~~~~~~~~~~~~~~~~~~~~~~~~~~~~~~~~~~~~~~~~~~~~~~~~~~~~~~~
2481  TCAGATTCTG GTAATAAATC CATCACTCGC GCTTCTCGTT TAATAAGTGA GTTTTTGGAA CCAATGGGTT TTGTTAAATG

      ~~~~~~~~~~~~~~~~~~~~~~~~~~~~~~~~~~~~~~~~~~~~~~~~~~~~~~~~~~~~~~~~~~~~~~~~~~~~~~~~~~~~~~~
2561  TAAAAAAATA AATAGTAAAT CAATGAGTAA TTATATACCT AAAAAAATTT TTTTAACTCC CATGTTTTTT ATGTTATGTG

      ~~~~~~~~~~~~~~~~~~~~~~~~~~~~~~~~~~~~~~~~~~~~~~~~~~~~~~~~~~~~~~~~~~~~~~~~~~~~~~~~~~~~~~~
2641  GTATTTCACC ATCAGAAATA AATCATTTTT TATCAAAAAA AATAAAACCA TTAAAAAAAT TAAAAAAACA AGAAAAAAGT

      ~~~~~~~~~~~~~~~~~~~~~~~~~~~~~~~~~~~~~~~~~~~~~~~~~~~~~~~~~~~~~~~~~~~~~~~~~~~~~~~~~~~~~~~
2721  GCATTTATTT CTTTTACAGA TATGAAGATT ATATCGCAAT TAGATGAAAG ATCAGCTAGA ACAAAAATTT TAAATGCTTT

      ~~~~~~~~~~~~~~~~~~~~~~~~~~~~~~~~~~~~~~~~~~~~~~~~~~~~~~~~~~~~~~~~~~~~~~~~~~~~~~~~~~~~~~~
2801  AATTAATTAT TACACAGCTA GTGAATTAAC AAAAATAGGT CCGAAGGGTT TAAAAAAAAA GATAGATATT GAATATAGTA
                                                              SL4
                                       ~~~~~~~~~~~~~~~~~~~~~~~~~~~~~~~~~~~~~~~~~~~~~~~~~~~~~
                    Prom. 4
       ~~~~~~~~~~~~~~~~~~~~~~~~~~~~~~~~~~~~~~~~~~~~~
      ~~~~~~~~~~~~~~~~~~~~~~~~~~~~~~~~~~~
2881  ATTTATGTAA TTTATATAAA AAAAAATCAT AAAATAAATT TATACAATAA AAATTTCATG AGACATCATA TACAAAATTA
                                                                             RBS 3
      ~~~~~~~~~~~~~~~~~~~~~~~~~~~~~~~~~~~~~~~~~~~~~~~~~~~                    ~~~~~~
2961  TTTTGTATAT GATGTCTTAT GAAATTTTTA TTGTATAAAT TTATTTTTAT CCAATAAAAC ATTCCATTAC CTTAATTTGG
                                       leuA (3051- 4610)
                 ~~~~~~~~~~~~~~~~~~~~~~~~~~~~~~~~~~~~~~~~~~~~~~~~~~~~~~~~~~~~~~~~~~~~~~~~~~~~~
3041  AAAAATTTTT GTGAAATCTA AAGTTGTTAT TTTTGATACC ACGCTACGCG ATGGAGAACA AGCATTACAA GCAAGTCTTA

      ~~~~~~~~~~~~~~~~~~~~~~~~~~~~~~~~~~~~~~~~~~~~~~~~~~~~~~~~~~~~~~~~~~~~~~~~~~~~~~~~~~~~~~~
3121  GTGTTAAAGA AAAATTACAA ATTGCACTAT CTTTAGAAAA ATGTGGGATA GACATTTTAG AAATAGGATT TCCTGTTTCA

      ~~~~~~~~~~~~~~~~~~~~~~~~~~~~~~~~~~~~~~~~~~~~~~~~~~~~~~~~~~~~~~~~~~~~~~~~~~~~~~~~~~~~~~~
3201  TCACCCGGAG ATTTTAAATC AGTTCAAACT ATATCTAAAA ATATTAAAAA TAGTCGAATA TGTAGTTTAG CTCGTTGTAT

      ~~~~~~~~~~~~~~~~~~~~~~~~~~~~~~~~~~~~~~~~~~~~~~~~~~~~~~~~~~~~~~~~~~~~~~~~~~~~~~~~~~~~~~~
3281  AGAAAAAGAC ATCGATGCAG CCGGAGAAGC TATGTCTTCA TCTGATTCTT TTCGAATTCA TATTTTTTTA GCTACTTCAA

      ~~~~~~~~~~~~~~~~~~~~~~~~~~~~~~~~~~~~~~~~~~~~~~~~~~~~~~~~~~~~~~~~~~~~~~~~~~~~~~~~~~~~~~~
3361  CACTTCATAT GGAATCTAAA TTAAAGAAAA ATTTTAACGA AATAATAGAT ATGGCTGTTT TTTCAGTAAA AAAAGCCTTA

      ~~~~~~~~~~~~~~~~~~~~~~~~~~~~~~~~~~~~~~~~~~~~~~~~~~~~~~~~~~~~~~~~~~~~~~~~~~~~~~~~~~~~~~~
3441  CGTTATACTG ATGATATTGA ATTTTCTTGT GAAGATGCTA CTAGAACTAC AATGGATAAT TTATGTCGTA TTGTAGAAAC

      ~~~~~~~~~~~~~~~~~~~~~~~~~~~~~~~~~~~~~~~~~~~~~~~~~~~~~~~~~~~~~~~~~~~~~~~~~~~~~~~~~~~~~~~
3521  ATTAATTAAA TCAGGTGTGA AAACAATTAA TATCCCCGAT ACAGTAGGAT ATACTGTACC CAACGAATTA TCTTGTATAA

      ~~~~~~~~~~~~~~~~~~~~~~~~~~~~~~~~~~~~~~~~~~~~~~~~~~~~~~~~~~~~~~~~~~~~~~~~~~~~~~~~~~~~~~~
```

```
3601   TAAAAAATTT GCTTGAAAGA GTACCTAACA TTCATAAATC TATAATTTCA GTTCATTGTC ATGACGATTT AGGCATGGCT

       ~~~~~~~~~~~~~~~~~~~~~~~~~~~~~~~~~~~~~~~~~~~~~~~~~~~~~~~~~~~~~~~~~~~~~~~~~~~~~~~~~~~~~~~
3681   GTGGGAAATT CAATATCAGC TATACAGGCA GGTGCTAGAC AAATAGAAGG GACTATAAAC GGAATTGGCG AAAGAGCTGG

       ~~~~~~~~~~~~~~~~~~~~~~~~~~~~~~~~~~~~~~~~~~~~~~~~~~~~~~~~~~~~~~~~~~~~~~~~~~~~~~~~~~~~~~~
3761   TAATACAGCA TTAGAAGAAA TAATTATGGC TATAAAAGTA AGAGAAGATA TCTTAAGTGT TTCCACTAAT ATTAATTATA

       ~~~~~~~~~~~~~~~~~~~~~~~~~~~~~~~~~~~~~~~~~~~~~~~~~~~~~~~~~~~~~~~~~~~~~~~~~~~~~~~~~~~~~~~
3841   AAGAAATTTA TCGAACTAGT AAAATTGTTA GTCAGATTTG TAATATGCCA ATCCCATCTA ATAAAGCTAT AGTAGGTAGT

       ~~~~~~~~~~~~~~~~~~~~~~~~~~~~~~~~~~~~~~~~~~~~~~~~~~~~~~~~~~~~~~~~~~~~~~~~~~~~~~~~~~~~~~~
3921   AATGCGTTTG CACATTCTTC AGGTATCCAC CAAGATGGTG TATTAAAAAA TAGAAAAAAT TATGAAATTA TGGAACCAAG

       ~~~~~~~~~~~~~~~~~~~~~~~~~~~~~~~~~~~~~~~~~~~~~~~~~~~~~~~~~~~~~~~~~~~~~~~~~~~~~~~~~~~~~~~
4001   TAGTATTGGT TTAAAAGAAG TAAAACTTAA CTTAACCTCT CGTTCTGGAA GAGCAGCAGT AAAACATTAT ATGGATGAAA

       ~~~~~~~~~~~~~~~~~~~~~~~~~~~~~~~~~~~~~~~~~~~~~~~~~~~~~~~~~~~~~~~~~~~~~~~~~~~~~~~~~~~~~~~
4081   TGGGTTATAA TAATAGTGAT TATAATATAG ATGAACTTTA TATTGCTTTT TTAAAGTTAG CAGATAAAAA AGGTCAGGTT

       ~~~~~~~~~~~~~~~~~~~~~~~~~~~~~~~~~~~~~~~~~~~~~~~~~~~~~~~~~~~~~~~~~~~~~~~~~~~~~~~~~~~~~~~
4161   TTTGACTATG ATTTAGAAGC ATTAGCTTTT ATTAATAAAC AACAAGATGA ATGGGAATAC TTTTCTTTAA AATTTTTTAG

       ~~~~~~~~~~~~~~~~~~~~~~~~~~~~~~~~~~~~~~~~~~~~~~~~~~~~~~~~~~~~~~~~~~~~~~~~~~~~~~~~~~~~~~~
4241   TGTGCAGTCT ATTTCTAATA GTTTATCTAC TGCATCAGTA AAATTATTAT GTGGCAAAAA AACATACACA GAATCTTCTA
                                                           Prom. 5
                                                ~~~~~~~~~~~~~~~~~~~~~~~~~~~~~~~~~~~~~~~~
       ~~~~~~~~~~~~~~~~~~~~~~~~~~~~~~~~~~~~~~~~~~~~~~~~~~~~~~~~~~~~~~~~~~~~~~~~~~~~~~~~~~~~~~~
4321   CTACAAGCAA TGGACCAGTT GATGCTATTT ACCAAGCATT AAATAGAATT GCATGTTTTC CTATTATATT ACAAAAATTT

       ~~~~~~~~~~~~~~~~~~~~~~~~~~~~~~~~~~~~~~~~~~~~~~~~~~~~~~~~~~~~~~~~~~~~~~~~~~~~~~~~~~~~~~~
4401   CAACTTGTAG CTAAGGGGAA GGGTAAAGAT GCGTTAGGTC AAGTAGATAT CTTGGTTGAA CATAAAAAAA GGAAGTTTCA

       ~~~~~~~~~~~~~~~~~~~~~~~~~~~~~~~~~~~~~~~~~~~~~~~~~~~~~~~~~~~~~~~~~~~~~~~~~~~~~~~~~~~~~~~
4481   TGGAGTGGGT TTGGCTACTG ATATTATTGA AGCATCAGCT CAAGCAATGA TCAATGTACT AAATAACATA TGGAAAGCAA
                                                                                         RBS 4
       ~~~~~~~~~~~~~~~~~~~~~~~~~~~~~~~~~~~~~~~~~~~~~~~~~~~~~~                             ~~~~
4561   AACAAGTTAA CAAAAAATTA AAAATTTTAA AAGATTTTAA AAAAAAATAA TACATCTAGA TATAGAAATA TAATATTTTT
                                                       leuB (4671- 5762)
       ~                                ~~~~~~~~~~~~~~~~~~~~~~~~~~~~~~~~~~~~~~~~~~~~~~~~~~~~~~
4641   TGTTGATTTT TATTAAAGAG AAAAATTTTT ATGAAAAAAA ATTATCGTAT CGCTGTATTA TCTGGTGATG GAATAGGTCC
                                                         SL5
                                                   ~~~~~~~~~~~~~~~~~
                                               Term. 5
                                           ~~~~~~~~~~~~~~~~
       ~~~~~~~~~~~~~~~~~~~~~~~~~~~~~~~~~~~~~~~~~~~~~~~~~~~~~~~~~~~~~~~~~~~~~~~~~~~~~~~~~~~~~~~
4721   TGAAGTCATG CAAGAAGCAT GTAAAATTTT AAATGTTTTA AAAAAATATT TTTTTCTATC TTTAGAAATA CAAAAATTTA

       ~~~~~~~~~~~~~~~~~~~~~~~~~~~~~~~~~~~~~~~~~~~~~~~~~~~~~~~~~~~~~~~~~~~~~~~~~~~~~~~~~~~~~~~
4801   ATATTGGTGG TATAGCTATT GAACGTGAAG GTGTTGCTTT GCCAAAAACT ACATTGCTAG GATGTGAAAA TTCTGATTCA

       ~~~~~~~~~~~~~~~~~~~~~~~~~~~~~~~~~~~~~~~~~~~~~~~~~~~~~~~~~~~~~~~~~~~~~~~~~~~~~~~~~~~~~~~
4881   ATTTTATTAG GTTCTGTAGG TGGTAAGAAG TGGGATAACC TTCCAGTAGA ACAACGTCCT GAAAGAGCAG CTTTATTGCC

       ~~~~~~~~~~~~~~~~~~~~~~~~~~~~~~~~~~~~~~~~~~~~~~~~~~~~~~~~~~~~~~~~~~~~~~~~~~~~~~~~~~~~~~~
4961   TTTAAGAAAA TACTTTAATC TTTTTTCTAA TTTAAGACCA GCAAAATTAT ATCCAGAACT AAAATGTTTA TCACCTCTTC

       ~~~~~~~~~~~~~~~~~~~~~~~~~~~~~~~~~~~~~~~~~~~~~~~~~~~~~~~~~~~~~~~~~~~~~~~~~~~~~~~~~~~~~~~
5041   GTTCAGATAT TGTAAAAAAT GGTTTTGATA TATTATGTGT AAGAGAATTA ACAGGTGGAA TTTATTTTGG TGAACCTAAA

       ~~~~~~~~~~~~~~~~~~~~~~~~~~~~~~~~~~~~~~~~~~~~~~~~~~~~~~~~~~~~~~~~~~~~~~~~~~~~~~~~~~~~~~~
5121   GGCTTTGTAA ATAAAAACAA TACTAAATAT GCTTTTGATA CAGAAATTTA TCATGAGTAT GAAATTATCC GTATTGCTCA

       ~~~~~~~~~~~~~~~~~~~~~~~~~~~~~~~~~~~~~~~~~~~~~~~~~~~~~~~~~~~~~~~~~~~~~~~~~~~~~~~~~~~~~~~
5201   TTTAGCTTTT AAATTGGCAC GATCTAAGAA GAAGAAAGTC TGTTCTATAG ATAAATCAAA TGTTCTTCGA AGTTCTATTT

       ~~~~~~~~~~~~~~~~~~~~~~~~~~~~~~~~~~~~~~~~~~~~~~~~~~~~~~~~~~~~~~~~~~~~~~~~~~~~~~~~~~~~~~~
5281   TATGGGGAAA AGTAGTTGAA AGTGTTTCTA AAAAATACCC AGATGTTCAT TTATCTCATT TATATATTGA TAATGCTGCT

       ~~~~~~~~~~~~~~~~~~~~~~~~~~~~~~~~~~~~~~~~~~~~~~~~~~~~~~~~~~~~~~~~~~~~~~~~~~~~~~~~~~~~~~~
5361   ATGCAAATAA TTAAAGATCC TAATCAGTTT GATGTATTAT TATGCTCAAA TCTTTTTGGA GATATTATTT CTGATGAATG

       ~~~~~~~~~~~~~~~~~~~~~~~~~~~~~~~~~~~~~~~~~~~~~~~~~~~~~~~~~~~~~~~~~~~~~~~~~~~~~~~~~~~~~~~
5441   TGCTACAATT ACAGGTTCAA TTGGGATGTT ACCATCAGCA AGTTTTAATG AAAAAAATTT TGGATTATAT GAGCCGGCAG

       ~~~~~~~~~~~~~~~~~~~~~~~~~~~~~~~~~~~~~~~~~~~~~~~~~~~~~~~~~~~~~~~~~~~~~~~~~~~~~~~~~~~~~~~
```

```
5521  GCGGATCAGC ACCTGATATT GAAGGTAAAA ATATTGCTAA TCCGATTGCT CAAATACTTT CACTTTCTAT GTTAGTTAGA

      ~~~~~~~~~~~~~~~~~~~~~~~~~~~~~~~~~~~~~~~~~~~~~~~~~~~~~~~~~~~~~~~~~~~~~~~~~~~~~~~~~~~~~~~
5601  TATGGCATGA ATTTAAATCA GATAGCAGAT AAAATTGATA AAGCTGTTAA TAATGTATTA AAAAAAGGTT ATAGAACTTC
                                                                                     RBS 5
                                                                                     ~~~~~

      ~~~~~~~~~~~~~~~~~~~~~~~~~~~~~~~~~~~~~~~~~~~~~~~~~~~~~~~~~~~~~~~~~~~~~~~~~~~~~~~~~~~~~~~
5681  AGATATATCT CATGATAATA ATTTTTTAAA AACAGATGAA ATGGGTGATC TTATTGTTGA TTCTTTAATT AATGGTAAAT
                                      leuC (5764 - 7179)
         ~~~~~~~~~~~~~~~~~~~~~~~~~~~~~~~~~~~~~~~~~~~~~~~~~~~~~~~~~~~~~~~~~~~~~~~~~~~~~~~~~~~~
      ~~
5761  GACATGAAGA AAACATTATA CGATAAAATA TATGATTCAC ATATTGTTTA TGAAGAGAAA AATAATACAT CTCTTTTATA

      ~~~~~~~~~~~~~~~~~~~~~~~~~~~~~~~~~~~~~~~~~~~~~~~~~~~~~~~~~~~~~~~~~~~~~~~~~~~~~~~~~~~~~~~
5841  TATAGATTTA CATTTACTTC ATGAGGTTAC ATCACCTCAA GCCTTTGATT CATTACGAGA TAAAAATCGA AAGGTTAGAC

      ~~~~~~~~~~~~~~~~~~~~~~~~~~~~~~~~~~~~~~~~~~~~~~~~~~~~~~~~~~~~~~~~~~~~~~~~~~~~~~~~~~~~~~~
5921  AGCCTAAAAA AACTTTTGCT ACAATGGATC ATAATGTTTC GACAACTAGT CAAGATATTA ATGCTTCAGG TTCTATGGCA

      ~~~~~~~~~~~~~~~~~~~~~~~~~~~~~~~~~~~~~~~~~~~~~~~~~~~~~~~~~~~~~~~~~~~~~~~~~~~~~~~~~~~~~~~
6001  AAGGTACAAA TGCAAGAATT AATTAAAAAT TGTTCCGAGT TTAATATATC ATTATATGAT ATAAAAAATC CTAATCAAGG

      ~~~~~~~~~~~~~~~~~~~~~~~~~~~~~~~~~~~~~~~~~~~~~~~~~~~~~~~~~~~~~~~~~~~~~~~~~~~~~~~~~~~~~~~
6081  GATTGTTCAT GTTATTAGTC CTGAAAAAGG GATGACTTTG CCGGGTGTGA CTATTGTATG TGGTGACTCA CATACATCGA

      ~~~~~~~~~~~~~~~~~~~~~~~~~~~~~~~~~~~~~~~~~~~~~~~~~~~~~~~~~~~~~~~~~~~~~~~~~~~~~~~~~~~~~~~
6161  CGCATGGCGC ATTTGGAGCA TTATCTTTTG GTATTGATAC TTCAGAAGTT GAACATGTTC TTGCAACTCA AACATTAAAA

      ~~~~~~~~~~~~~~~~~~~~~~~~~~~~~~~~~~~~~~~~~~~~~~~~~~~~~~~~~~~~~~~~~~~~~~~~~~~~~~~~~~~~~~~
6241  CAACAGCGTT TTAAAAATAT GAAAATAGAA ATTACAGGAG AAATTCAAAA ATTTGTCACT GCTAAAGATT TGATTTTGTT

      ~~~~~~~~~~~~~~~~~~~~~~~~~~~~~~~~~~~~~~~~~~~~~~~~~~~~~~~~~~~~~~~~~~~~~~~~~~~~~~~~~~~~~~~
6321  TATTATTGGA AAATTAGGAT CATCTGGAGG TGCAGGATAT ATAATTGAAT TTTGTGGTAA TGTGATTGAA AAAATGAGCA

      ~~~~~~~~~~~~~~~~~~~~~~~~~~~~~~~~~~~~~~~~~~~~~~~~~~~~~~~~~~~~~~~~~~~~~~~~~~~~~~~~~~~~~~~
6401  TGGAAGAAAG GATGACAATT TGTAATATGG CAATTGAAAT AGGTGCAAAG TCAGGTTTGA TAGCACCAGA TGAAGTGACA
                 Term. 6
               ~~~~~~~~~~~~~~

      ~~~~~~~~~~~~~~~~~~~~~~~~~~~~~~~~~~~~~~~~~~~~~~~~~~~~~~~~~~~~~~~~~~~~~~~~~~~~~~~~~~~~~~~
6481  TTTTCTTATC TGAAAAATAA AATGTATGCA CCACGTGGTG TTTTTTGGAA AAAAGCATTA AATTTTTGGA AGAATTTGAA

      ~~~~~~~~~~~~~~~~~~~~~~~~~~~~~~~~~~~~~~~~~~~~~~~~~~~~~~~~~~~~~~~~~~~~~~~~~~~~~~~~~~~~~~~
6561  ATCTGATAAA AATGCTTTTT TTGATAAAGT TGTGAATATA AATATTTCTG ATCTCTCGCC ACAAATTACC TGGGGGACAA

      ~~~~~~~~~~~~~~~~~~~~~~~~~~~~~~~~~~~~~~~~~~~~~~~~~~~~~~~~~~~~~~~~~~~~~~~~~~~~~~~~~~~~~~~
6641  ATCCTGATCA GGTGATCTCA ATCGATCAAA AAATACCTGA TTTTAGTAGT TTTGATAATT TAATAAAGAA AGATTTAGCG

      ~~~~~~~~~~~~~~~~~~~~~~~~~~~~~~~~~~~~~~~~~~~~~~~~~~~~~~~~~~~~~~~~~~~~~~~~~~~~~~~~~~~~~~~
6721  AAATCTGCAT GTAAATATAT GGGTTTAAAA ATAGGTACTT ATTTAACTAA TATTACTGTT GATGAAGTTT TTATTGGATC

      ~~~~~~~~~~~~~~~~~~~~~~~~~~~~~~~~~~~~~~~~~~~~~~~~~~~~~~~~~~~~~~~~~~~~~~~~~~~~~~~~~~~~~~~
6801  TTGTACTAAT GGAAGAATAG AAGATTTAAG AGCTGCCTCA AAGATACTTA AAGATAAAAA AATTGCTAAT AATGTTAAAG

      ~~~~~~~~~~~~~~~~~~~~~~~~~~~~~~~~~~~~~~~~~~~~~~~~~~~~~~~~~~~~~~~~~~~~~~~~~~~~~~~~~~~~~~~
6881  CTATTGTTGT TCCTGGATCA AGTTCAGTTA AGAGAGAAGC TGAAAATGAA GGTTTAGATA AAATATTTAT TAATGCAGGA

      ~~~~~~~~~~~~~~~~~~~~~~~~~~~~~~~~~~~~~~~~~~~~~~~~~~~~~~~~~~~~~~~~~~~~~~~~~~~~~~~~~~~~~~~
6961  TTCGAATGGC GTTTACCTGG TTGTTCTATG TGTTTAGGTA TGAATAAAGA TAGATTAAAT GATGGTGAAC GTTGTGCGTC

      ~~~~~~~~~~~~~~~~~~~~~~~~~~~~~~~~~~~~~~~~~~~~~~~~~~~~~~~~~~~~~~~~~~~~~~~~~~~~~~~~~~~~~~~
7041  TACTAGCAAT CGAAATTTTG AGGGTCGCCA GGGGAGAGGG GGCGAACAC ATTTAGTAAG TCCTATTATG GCGGCTGCAG
                                                RBS 6
                                                ~~~~~             leuD (7182 - 7805)
      ~~~~~~~~~~~~~~~~~~~~~~~~~~~~~~~~~~~~~~~~~~~~~~~~~~~~~~~~~~~~~~~~~   ~~~~~~~~~~~~~~~~~~~~
7121  CTGCTGTATA CGGTAAATTT GTTGATGTTA GAAAATTATA TAATGGTGAG AATAATTAAA TATGTTTAAA TTTACTGGAC

      ~~~~~~~~~~~~~~~~~~~~~~~~~~~~~~~~~~~~~~~~~~~~~~~~~~~~~~~~~~~~~~~~~~~~~~~~~~~~~~~~~~~~~~~
7201  ATGCTGGTAT CGTTGTTCCT TTAGATATAT CTAATATAGA TACAGATATT ATTATTCCGA AGCAATTTTT GAAAAGAGTA

      ~~~~~~~~~~~~~~~~~~~~~~~~~~~~~~~~~~~~~~~~~~~~~~~~~~~~~~~~~~~~~~~~~~~~~~~~~~~~~~~~~~~~~~~
7281  AACAAAATTG GATTTGGTAA ATATTTATTT CATGACTGGC GTTTTATTGA TGCAAATCAA TTAGTAAAAA ATGAGGATTT

      ~~~~~~~~~~~~~~~~~~~~~~~~~~~~~~~~~~~~~~~~~~~~~~~~~~~~~~~~~~~~~~~~~~~~~~~~~~~~~~~~~~~~~~~
7361  TATACTCAAT AAAAAAATTT ATAAAAACGC AAGTATTTTA TTAACTAGAG AGAATTTTGG TTGCGGCTCA TCGAGAGAAC

      ~~~~~~~~~~~~~~~~~~~~~~~~~~~~~~~~~~~~~~~~~~~~~~~~~~~~~~~~~~~~~~~~~~~~~~~~~~~~~~~~~~~~~~~
```

```
7441  ATGCTGTTTG GTCTCTAGTA GACTATGGAT TCAAAGTAAT AATTGCACCT AGTTTTGCTG ATATTTTTTA TAATAATAGT

      ~~~~~~~~~~~~~~~~~~~~~~~~~~~~~~~~~~~~~~~~~~~~~~~~~~~~~~~~~~~~~~~~~~~~~~~~~~~~~~~~~~~~~~~
7521  TTTAACAATA AGCTTCTTTT AATCACTTTA AGTTCGAGTG AAATTACTTT TTTATTTGAT ATTGTAAAAA ATAATATAGG

      ~~~~~~~~~~~~~~~~~~~~~~~~~~~~~~~~~~~~~~~~~~~~~~~~~~~~~~~~~~~~~~~~~~~~~~~~~~~~~~~~~~~~~~~
7601  TATTACTTTT GATGTAAGTT TAGTCGAAAA AACGGTTACT GTAAATAAGG AAGTATTTTC ATTTGAATTA GATGATTTTC

      ~~~~~~~~~~~~~~~~~~~~~~~~~~~~~~~~~~~~~~~~~~~~~~~~~~~~~~~~~~~~~~~~~~~~~~~~~~~~~~~~~~~~~~~
7681  ATTACTTTTG TTTGTTAAAT GATTTAGATA ATATTGATTT GACTATGAAG CATTTATCAG AAATAAAATC TTATGAAAGT

      ~~~~~~~~~~~~~~~~~~~~~~~~~~~~~~~~~~~~~~~~~~~~~~~~~
7761  CGTATATCTG ATTTTTTATT AGAAAGAAGA GATTTCCAAT CTTAA
```

Figure S3. Comparative evolutionary analysis of pLeu promoters in the Aphididae family.
The analysis was performed on ten aphid species belonging to two tribes of the Aphididae family which diverged more than 50 Myr ago: the Macrosiphini (*Acyrthosiphon pisum*, *Aulacorthum solani*, *Macrosiphum rosae*, *Metopolophium dirhodum*, *Uroleucon sonchi* and *Diuraphis noxia*) and the Aphidini (*Schizaphis graminum*, *Rhopalosiphum cerasifoliae*, *Rhopalosiphum insertum* and *Rhopalosiphum padi*). We were able to locate: (i) the *repA2* promoter in 9/10 species (mean LDF ± SE = -12.0 ± 1.6); we identified however the *M. rosae* boxes by visual homology, the negative BPROM resulting from a one-base pair difference thresholding effect; (ii) the *leuA* promoter in 10/10 species (mean LDF ± SE = -9.6 ± 0.44); (iii) the *yqhA* and *repA1* promoters in 4/4 species (mean LDF ± SE being -16.2 ± 3.0 for *yqhA* and -14.2 ± 0.9 for *repA1*); and (iv) for *leuB*: the alternate promoter signal was located as a conserved internal signal to *leuA* cds for two *Macrosiphini* species, whilst only one potential signal was found for the *Aphidini* species, immediately upstream of the *leuB* cds in *R. padi*. All these *leuB* signals were the weakest found with BPROM, whether included, or not, in coding regions (LDF = -6.2 ± 1.0).

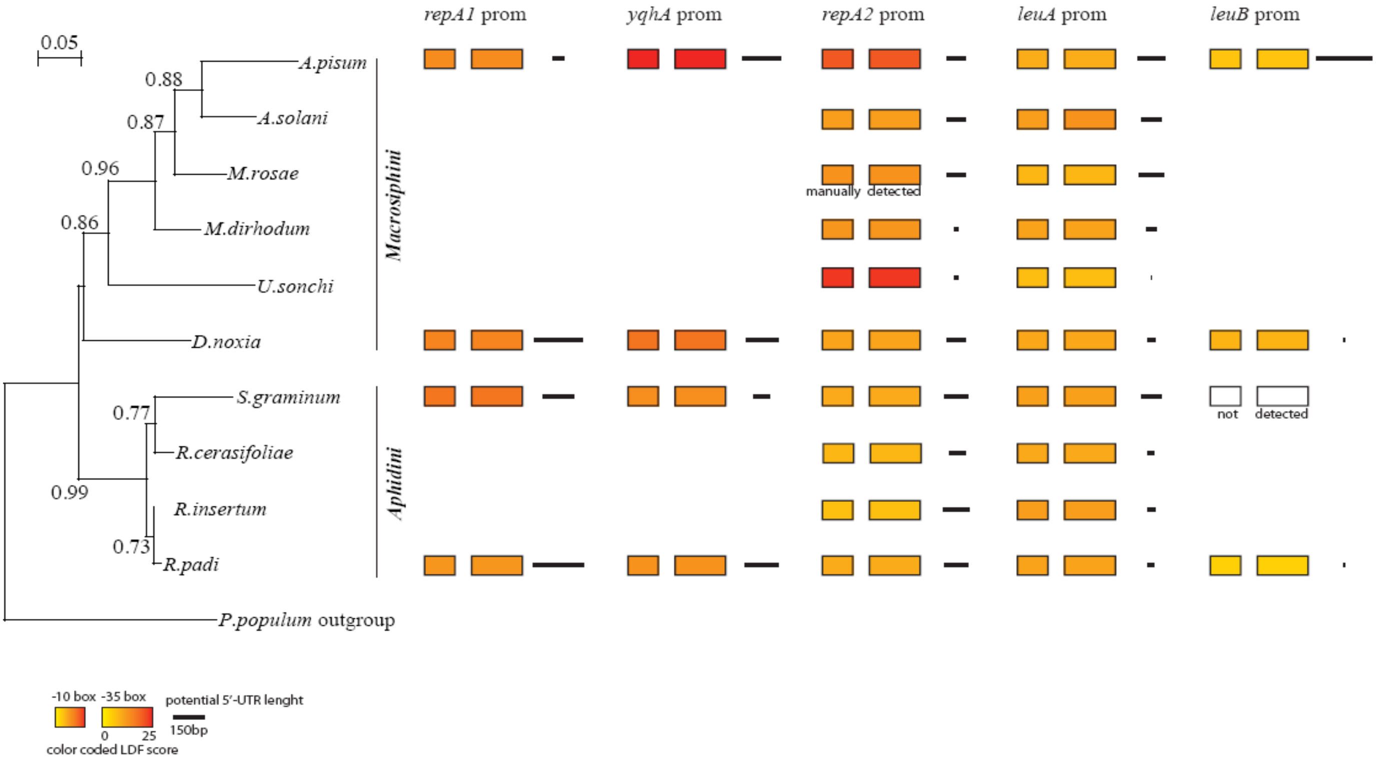

Table S1. Neosynthesis of amino acids from the carbons of sucrose in the symbiotic pea aphid reared on diets with leucine concentrations ranging from 0 to 80 mM.
Values (means ± SE, n = 5) are expressed in sucrose equivalent (nmoles) per unit of aphid fresh weight (mg).

Amino acid	Dietary leucine concentration					*P*-value *
	0 mM	20 mM	40 mM	60 mM	80 mM	
Aspartic acid	9.1 ± 0.6	9.9 ± 0.5	10.1 ± 0.3	10.1 ± 0.4	10.2 ± 0.4	0.49
**Threonine**	**2.4 ± 0.4 [a]**	**2.8 ± 0.2 [a]**	**3.6 ± 0.2 [b]**	**4.3 ± 0.2 [b]**	**3.9 ± 0.3 [b]**	**< 0.001**
Serine	5.7 ± 0.3	5.9 ± 0.1	6.3 ± 0.3	6.8 ± 0.3	6.4 ± 0.5	0.22
Glutamic acid	18.8 ± 0.9	17.8 ± 0.7	18.3 ± 0.6	17.5 ± 1.0	18.5 ± 0.5	0.77
Proline	10.4 ± 0.2	12.0 ± 0.4	11.8 ± 0.5	11.6 ± 0.5	12.3 ± 0.6	0.14
Glycine	4.2 ± 0.2	3.9 ± 0.1	3.9 ± 0.3	4.1 ± 0.2	4.1 ± 0.1	0.77
Alanine	13.6 ± 0.9	13.9 ± 0.5	14.5 ± 0.5	14.8 ± 1.1	15.8 ± 0.2	0.21
**Valine**	**5.6 ± 0.2 [a]**	**6.8 ± 0.2 [b]**	**8.2 ± 0.2 [c]**	**8.9 ± 0.6 [c]**	**9.0 ± 0.7 [c]**	**< 0.001**
**Cystine**	**0.7 ± 0.1 [a]**	**0.7 ± 0.1 [a]**	**0.3 ± 0.05 [b]**	**0.5 ± 0.05 [ab]**	**0.5 ± 0.1 [ab]**	**0.02**
Methionine	0.6 ± 0.1	0.5 ± 0.1	0.5 ± 0.2	0.8 ± 0.1	0.6 ± 0.1	0.25
Isoleucine	5.9 ± 0.4	4.4 ± 0.2	5.3 ± 0.3	5.4 ± 0.6	5.1 ± 0.3	0.14
**Leucine**	**19.7 ± 1.4 [a]**	**6.7 ± 0.6 [b]**	**4.8 ± 0.3 [c]**	**3.3 ± 0.4 [cd]**	**2.4 ± 0.3 [d]**	**< 0.001**
**Tyrosine**	**3.4 ± 0.3 [a]**	**3.2 ± 0.3 [a]**	**5.0 ± 0.3 [b]**	**5.2 ± 0.2 [b]**	**5.2 ± 0.7 [b]**	**0.002**
Phenylalanine	6.4 ± 0.3	6.2 ± 0.2	7.0 ± 0.2	7.0 ± 0.5	7.4 ± 0.5	0.12
**Lysine**	**3.8 ± 0.4 [a]**	**3.4 ± 0.2 [a]**	**4.8 ± 0.2 [b]**	**4.9 ± 0.6 [b]**	**5.4 ± 0.4 [b]**	**0.006**
Histidine	nd †	nd	nd	nd	nd	
Arginine	nd	nd	nd	nd	nd	

* *P*-values of ANOVA (diet effect) performed for each amino acid. When the diet effect was significant (bold values, F-test *P*-value < 0.05), means were further compared with Student-Newman-Keuls test; columns with different letters are significantly different.
† nd = null value or below the detection threshold.

Table S2. Sequence information on primers used for real-time quantitative PCR and operon validation experiments.

For each couple of primers, sequences, lengths, annealing temperature and amplicon length are specified.

Gene	Primer	Sequence primer 5'- 3'	Primer length (in bp)	Primer Ta (in °C)	Amplicon length (in bp)
atpA	atpA-For	AAATGATTTTATTGCCTAA	19	47.1	99
	atpA-Rev	CAGAAATATGAATATAGGGA	20		
ilvH	ilvH-For	GAATCAGGTGCACTATCAAG	20	46.8	90
	ilvH-Rev	AGGATCTTCAGTAGGTGCTA	20		
ilvI	ilvI-For	TCTTTAATGGGATTAGGTAG	20	47.5	183
	ilvI-Rev	AACAATCGCATTTGG	15		
leuA	leuA-For	GATATACTGTACCCAACGAA	20	46.8	178
	leuA-Rev	ATTCCGTTTATAGTCCCT	18		
leuB	leuB-For	TTGCCAAAAACTACATTGC	19	47.1	93
	leuB-Rev	TTCTACTGGAAGGTTATCCC	20		
leuC	leuC-For	AGCCTTTGATTCATTACGAG	20	47.4	127
	leuC-Rev	TACCTTTGCCATAGAACCT	19		
leuD	leuD-For	ACATGCTGGTATCGTTGTTC	20	44.6	125
	leuD-Rev	AACGCCAGTCATGAA	15		
leuABCD	ABCD-For	TAAACGGAATTGGCGAAAGA	20	50.8	3697
	ABCD-Rev	GATGAGCCGCAACCAAA	17		
repA1	repA1-For	ATGGGTTTTGTTACTTGC	18	46.4	154
	repA1-Rev	TATTGATCCACCCTAATTGT	20		
repA2	repA2-For	ATTATATCGCAATTAGATGA	20	43.6	105
	repA2-Rev	TAAACCCTTCGGACCT	16		
rplX	rplX-For	AAAAAGAAGCACCTATTCAT	20	44.4	93
	rplX-Rev	TCCCTTCTTCAAACCTAA	18		
rpmC	rpmC-For	GGCATTGGTAGAATTTAGAA	20	46.8	184
	rpmC-Rev	CTCTTCCTTTGAATCTAGCA	20		
yqhA	yqhA-For	TTATCATGCCAGATATTGTG	20	47	174
	yqhA-Rev	CCATCCACCCTAATCTTT	18		

Table S3. Descriptive statistics from the BestKeeper software of the three candidates for data normalization, the *atpA*, *rplx* and *rpmC* genes.

On the three tested genes, only *atpA* and *rplX* met the criteria imposed by the Bestkeeper analysis: standard deviation ≤ 1 CP between the three tested conditions at each time point.

Abbreviations: n: number of samples; geo Mean [CP]: the geometric mean of CP; ar Mean [CP]: the arithmetic mean of CP; Min [CP] and Max [CP]: the extreme values of CP; std dev [± CP]: the standard deviation of the CP; CV [% CP]: the coefficient of variance expressed as a percentage on the CP level. Conditions showing significant gene variation (Cp>>1) are in red.

	**12 hours**			**1 day**			**2 days**			**3 days**			**7 days**		
	atpA	***rplX***	***rpmC***	***atpA***	***rplX***	***rpmC***	***atpA***	***rplX***	***rpmC***	***atpA***	***rplX***	***rpmC***	***atpA***	***rplX***	***rpmC***
**n**	9	9	9	9	9	9	9	9	9	9	9	9	9	9	9
**geo Mean [CP]**	25.90	26.30	26.27	24.64	24.41	24.86	24.88	21.44	25.65	24.65	21.68	24.83	25.92	23.54	23.64
**ar Mean [CP]**	25.92	26.32	26.46	24.65	24.43	24.90	24.91	21.47	25.76	24.67	21.71	24.94	25.94	23.54	23.65
**min [CP]**	24.77	24.8	23.68	23.78	23.33	22.77	23.5	19.94	22.68	22.74	20.31	21.33	24.7	22.77	22.81
**max [CP]**	27.55	28.04	31.44	25.72	26.41	26.67	26.49	23.62	28.34	25.75	23.45	27.17	27.9	24.27	24.65
**std dev [± CP]**	1.01	0.98	3.02	0.67	1.07	1.22	0.99	0.89	2.07	1.05	0.98	2.13	0.92	0.49	0.58
**CV [% CP]**	3.89	3.73	11.42	2.70	4.37	4.90	3.97	4.15	8.03	4.25	4.53	8.54	3.56	2.09	2.43

Table S4. Overall transcriptional response of *B. aphidicola* to variations of leucine concentration in the aphid diet.

CP values are indicated for each gene and for each sample. A sample is one of the three independent biological replicates for each condition (0, 20 or 60 mM leucine) and at each time point (12 h, 1, 2, 3 or 7 days) used in this experiment. For each sample, total RNA was extracted independently and 1 µg of it was retro-transcribed and then used for the quantitative PCR reaction. For each gene, the PCR efficiency represents the mean of all the reaction efficiencies obtained in this study.

		**Gene**	***atpA***	***rplX***	***rpmC***	***ilvI***	***ilvH***	***leuA***	***leuB***	***leuC***	***leuD***	***repA1***	***repA2***	***yqhA***
		**PCR efficiency**	**1.78**	**1.99**	**1.86**	**1.93**	**1.92**	**1.85**	**2.00**	**1.98**	**1.82**	**1.76**	**1.81**	**1.73**
**12h**	**20 mM**	Sample A1	25.64	25.75	23.87	19.83	20.90	25.25	23.81	21.78	23.55	28.05	29.84	27.69
		Sample A2	25.95	26.66	24.77	20.13	20.25	25.88	24.50	20.99	23.27	29.43	29.67	26.14
		Sample A3	24.83	24.80	24.34	19.49	20.50	25.72	22.18	21.50	22.94	27.56	29.79	29.23
	**0 mM**	Sample B1	24.93	27.68	24.08	18.96	21.76	24.02	22.01	19.45	21.96	28.67	27.95	23.78
		Sample B2	24.77	27.31	24.43	18.17	20.73	24.23	22.27	19.71	22.33	29.23	27.85	24.43
		Sample B3	24.92	28.04	23.68	19.01	20.44	24.26	21.46	19.57	22.43	28.06	27.76	23.13
	**60 mM**	Sample C1	27.34	25.53	31.01	26.30	22.86	29.97	27.87	25.41	26.88	37.23	34.65	30.92
		Sample C2	27.55	25.51	30.53	26.59	22.81	29.13	26.91	24.49	26.80	35.21	35.28	32.50
		Sample C3	27.41	25.59	31.44	28.06	23,00	29.38	26.79	24.78	26.91	36.20	33.98	29.33
			atpA	***rplX***	***rpmC***	***ilvI***	***ilvH***	***leuA***	***leuB***	***leuC***	***leuD***	***repA1***	***repA2***	***yqhA***
**D1**	**20 mM**	Sample A4	25.72	23.33	26.67	24.67	23.56	26.84	24.54	23.69	24.80	31.36	28.34	26,00
		Sample A5	25.52	23.68	26.43	24.53	23.67	27.36	24.57	23.65	25.08	30.97	29.43	25.96
		Sample A6	25.65	24.02	26.19	24.77	23.62	27.26	24.58	23.49	25.17	31.71	30.94	25.91
	**0 mM**	Sample B4	23.78	26.41	22.77	17.69	20.13	22.25	20.42	18.95	21.97	25.89	25.29	19.91
		Sample B5	24.01	26.01	23.62	17.11	19.04	22.65	20.32	19.20	21.17	26.30	25.52	18.95
		Sample B6	24.71	25.69	22.83	17.49	19.84	22.89	20.39	18.79	21.21	25.43	25.69	19.70
	**60 mM**	Sample C4	24.03	23.59	25.33	26.68	26.54	26.90	24.31	22.66	24.12	33.93	34.05	26.49
		Sample C5	24.23	23.65	25.21	27.96	26.93	27.89	25.12	23.28	24.83	33.32	33.63	26.58
		Sample C6	24.20	23.53	25.08	27.02	26.68	28.94	23.45	22.30	24.93	33.30	34.42	26.48
			atpA	***rplX***	***rpmC***	***ilvI***	***ilvH***	***leuA***	***leuB***	***leuC***	***leuD***	***repA1***	***repA2***	***yqhA***
**D2**	**20 mM**	Sample A7	25.98	22.09	28.34	22.41	21.86	28.23	22.84	21.17	24.90	31.83	31.49	34.33
		Sample A8	26.49	21.90	28.33	21.05	22.47	28.27	22.57	21.92	24.66	31.06	31.28	34.94
		Sample A9	26.46	22.23	28.28	23.73	22.47	28.05	22.73	21.55	24.93	31.47	31.65	33.71
	**0 mM**	Sample B7	23.50	23.62	22.68	16.31	18.47	21.38	20.85	18.63	21.10	25.88	23.68	19,00
		Sample B8	23.72	21.43	22.81	16.75	18.16	21.68	20.53	19.29	21.53	25.81	24.67	19.40
		Sample B9	24.06	21.53	22.80	17.67	18.44	21.67	20.73	18.54	21.31	26.48	24.75	19.79
	**60 mM**	Sample C7	24.52	20.46	25.42	21.68	20.64	25.93	22.30	21.58	23.22	28.11	30.25	24.04
		Sample C8	24.28	19.94	26.83	21.58	20.94	26.54	23.59	22.07	22.96	28.27	31.19	23.45
		Sample C9	25.15	20.04	26.31	21.74	20.92	26.69	23,00	21.58	23.39	28.59	29.92	22.88
			atpA	***rplX***	***rpmC***	***ilvI***	***ilvH***	***leuA***	***leuB***	***leuC***	***leuD***	***repA1***	***repA2***	***yqhA***
**D3**	**20 mM**	Sample A10	25.26	21.84	26.18	23.65	21.97	26.07	24.72	21.85	24.59	28.91	28.03	28.80
		Sample A11	25.51	21.36	26.65	22.73	22.88	26.76	25.12	21.90	25,00	28.20	28.64	27.91
		Sample A12	25.64	20.83	25.70	23.73	22.58	26.61	25.47	22.90	24.80	29.58	28.66	29.69
	**0 mM**	Sample B10	23.13	23.15	21.33	18.16	18.78	22.46	24.07	19.35	21.58	26.21	26.15	20.80
		Sample B11	23.43	22.81	22.14	18.31	18.90	23.06	22.21	19.80	21.82	26.23	27.17	19.89
		Sample B12	22.74	23.45	21.76	20.20	19.21	23.21	22.46	19.53	22.17	26.20	26.89	20.03
	**60 mM**	Sample C10	25.13	20.31	26.94	22.85	22.44	27.74	23.64	23.23	24.43	29.62	31.12	24.13
		Sample C11	25.75	20.94	26.56	22.45	22.31	27.99	23.67	23.10	24.78	29.98	31.35	24.38
		Sample C12	25.46	20.67	27.17	22.38	22.54	28.79	23.79	23.33	24.63	30.28	31.30	24.17
			atpA	***rplX***	***rpmC***	***ilvI***	***ilvH***	***leuA***	***leuB***	***leuC***	***leuD***	***repA1***	***repA2***	***yqhA***
**D7**	**20 mM**	Sample A13	25.71	23.64	23.57	23.36	24.34	24.09	24.59	24.14	24.16	25.66	26.02	23.85
		Sample A14	25.62	23.62	23.56	23.58	24.50	24.13	23.86	24.11	24.13	25.84	26.05	23.98
		Sample A15	25.99	23.49	23.73	23.56	24.45	24.21	24.22	24.10	24.06	26.06	25.77	24.32
	**0 mM**	Sample B13	24.70	22.77	22.81	22.89	23.95	23.55	23.64	23.58	23.66	25.02	25.28	23.71
		Sample B14	24.78	22.81	22.84	22.84	23.99	23.53	23.30	23.64	23.58	25,00	25.32	23.45
		Sample B15	24.74	22.89	22.87	22.92	23.92	23.62	23.15	23.56	23.62	25.07	25.32	23.33
	**60 mM**	Sample C13	26.76	24.19	24.51	24.50	25.06	24.71	25.22	24.56	24.70	26.45	26.38	24.89
		Sample C14	27.26	24.20	24.65	24.13	25.03	24.88	25.07	24.73	24.66	26.51	26.22	25.32
		Sample C15	27.90	24.27	24.29	24.52	25.02	24.91	24.86	24.87	24.54	26.66	26.36	25.28
